# Experimental and Computational ^77^Se NMR Spectroscopic Study on Selenaborane Cluster Compounds

**DOI:** 10.1021/acs.inorgchem.4c01890

**Published:** 2024-08-20

**Authors:** Jonathan Bould, Michael G. S. Londesborough, Oleg L. Tok

**Affiliations:** Institute of Inorganic Chemistry of the Czech Academy of Sciences, Husinec-Řež 250 68, Czech Republic

## Abstract

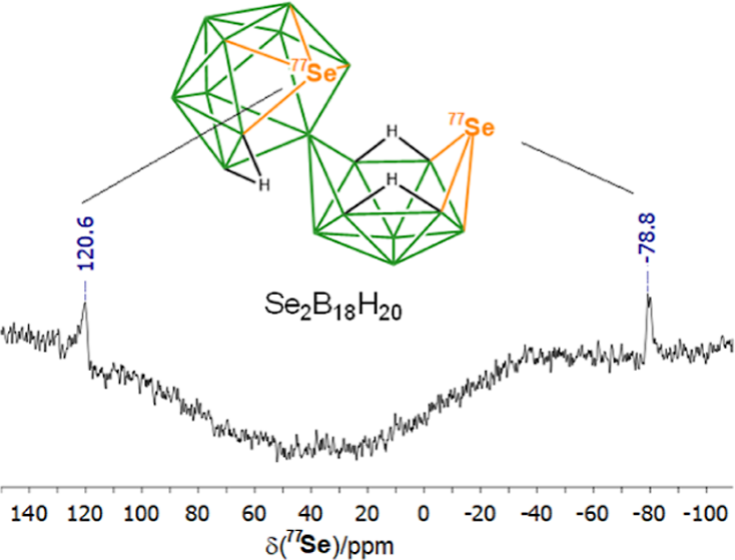

Calculated and measured ^77^Se nuclear magnetic resonance (NMR) chemical shift data on a diverse collection of 13 selenaborane cluster compounds, containing a total of 19 selenium centers, reveals a correlation between chemical shifts and the intracluster coordination of selenium atoms within their borane frameworks. A plot of the measured against calculated ^77^Se NMR chemical shifts shows an approximately linear relationship that can serve as a predictive tool in assessing the chemical shift range in which a selenium vertex from a particular compound might be expected to be found, thereby reducing expensive experimental time. Furthermore, the relative chemical shifts between selenium vertices in clusters containing more than one selenium atom are consistent across the range, thus allowing the assignment of the selenium resonances with a high degree of confidence even in relatively low-level density functional theory calculations. A new macropolyhedral 20-vertex selenaborane Se_2_B_18_H_20_ (**A**) is also reported.

## Abstract 2

Calculated ^77^Se chemical shifts are correlated with measured ^77^Se NMR chemical shifts for a series of macropolyhedral selenaboranes and related selenium containing boron compounds with up to 3 selenium atoms. NMR techniques to determine the assignment of selenium resonances in large borane cluster species are not experimentally practical. The correlation enables the assignment of the selenium vertices to their cluster positions.

## Introduction

Selenium-77 nuclear magnetic resonance (NMR) spectroscopy plays a useful role in the characterization of compound in both organoselenium chemistry and bioorganic chemistry, providing insights into structure and compound identity.^1−3^ For example, it has proven useful in enabling bioorganic selenametabolite compounds to be individually identified by their ^77^Se chemical shifts, thus providing a ^77^Se ′fingerprint′.^4^ In inorganic carboranyl boron cluster compounds, the presence of selenium has been used to assess the relative basicity of carboranylphosphines via the P–Se coupling constants.^5^ We, however, are interested in selenium chemical shifts in selenaborane macropolyhedral cluster compounds and the use of quantum chemical calculations to predict their chemical shifts.

Binary boron hydride compounds (boranes), a group of inorganic polyhedral molecules comprising clusters of boron and hydrogen atoms, display a diversity of structures surpassed only by the hydrocarbons.^6,7^ Borane clusters may incorporate main group elements that subrogate cluster {HB} vertices, often leading to an alteration in the cluster properties.^8−10^ Examples are found where Group 14, 15, and 16 elements such as carbon,^10^ nitrogen,^9^ and the chalcogens sulfur, selenium, and tellurium have been incorporated in borane and metallaborane clusters.^11−18^ In the case of selenium and sulfur, these have also been combined in so-called macropolyhedral clusters, which are defined as “borane compounds that contain two or more cages, with individual cages that are joined or fused to each other with two or more atoms held in common”.^19^ The synthesis and characterization of a number of new anionic macropolyhedral selenaboranes have been described,^20,21^ and, more recently, the photophysical properties of their luminescent neutral conjugate acids together with other luminescent macropolyhedral thiaboranes have also been reported.^22^ In terms of NMR spectroscopy, these selenaborane compounds were characterized by their ^11^B and ^1^H spectra. The nuclear spin-half property of selenium-77, its 7.6% natural abundance, and gyromagnetic ratio of 19.071523 × 10^–7^ rad·s^–1^·T^–1^ allow an additional and easily available metric using a routine single-pulse sequence for the characterization of selenaborane clusters, which can potentially deliver additional structural information. Nevertheless, historically, the measurement of ^77^Se chemical shifts during the characterization of selenaborane species has not been routine,^17,23−30^ and it has thus hitherto received little attention, although a recent publication has described a similar approach to our work reported herein, with a pairing of measured and calculated ^77^Se NMR chemical shifts in a number of perchlorinated monoselenaborane clusters.^31^ There is also a number of compounds that contain both B–H units, chalcogens and metal units, such as Mo or Fe, in which ^77^Se chemical shifts have been measured, but they feature cubane-type or other structural motifs that do not resemble conventional borane clusters based usually on an icosahedron.^15,16,32^ They thus fall outside the scope of the work reported here.

In selenaborane compounds with only one selenium vertex, the assignment of the selenium resonance is clear. However, the assignments are not so straightforward in the macropolyhedral and other selenaborane species reported here, which contain two or three selenium atoms per molecule. There are several NMR techniques for assigning ^11^B and ^1^H resonances in borane clusters to their cluster positions, and they typically include different variations of 1D experiments (coupled, broad-band or selective decoupled, etc.) together with 2D methods such as ^1^H–^11^B HMQC (heteronuclear multiple-quantum correlation) and ^1^H–^11^B HSQC (heteronuclear single-quantum correlation) and ^11^B–^11^B-COSY (homonuclear correlation spectroscopy) methods. However, there are no 1D or 2D correlation NMR techniques suitable for assigning the positions of selenium vertices in heteroborane species holding more than one selenium atom. This is because both ^1^H and ^11^B resonances are too broad to observe selenium-77 satellites or to apply the correlation methods based on long-range ^1^H–^77^Se or ^11^B–^77^Se couplings, which would enable a direct assignment of the boron resonances adjacent to the selenium vertices. We have thus resorted to quantum chemical calculations to investigate the possibility of achieving a suitably close correlation between calculated and measured values in order to assign the selenium resonances to their positions in selenaboranes. Herein, we endeavor to collate and describe the measured and calculated ^77^Se NMR chemical shifts of this range of selenaboranes, limited to those in which selenium acts as a vertex in the borane cluster rather than, for example, an *exo*-polyhedral substituent replacing an *exo*-terminal hydrogen atom and also where selenium is the only cluster heteroelement in order to eliminate the complications that will ensue from the inclusion of, for example, heavier ligated transition metal moieties mentioned above. We show how these collective data can be used to predict the region in which to expect the ^77^Se NMR signals to be found in new selenaboranes. This approach has been used previously for correlations between ^11^B calculated and measured values^33^ and a range of organic and inorganic selenium compounds^34,35^ but has not previously been applied to selenaboranes. Measured ^77^Se resonances are very sensitive to the local surroundings such that they can be spread over a long range of ca. 3000 ppm,^36−38^ and thus they can be time-consuming to locate, especially in weaker samples. Consequently, there is utility in accurately calculating the potential chemical shift range in which a particular selenium atom might be expected to be found.

## Results and Discussion

**Chart 1:**
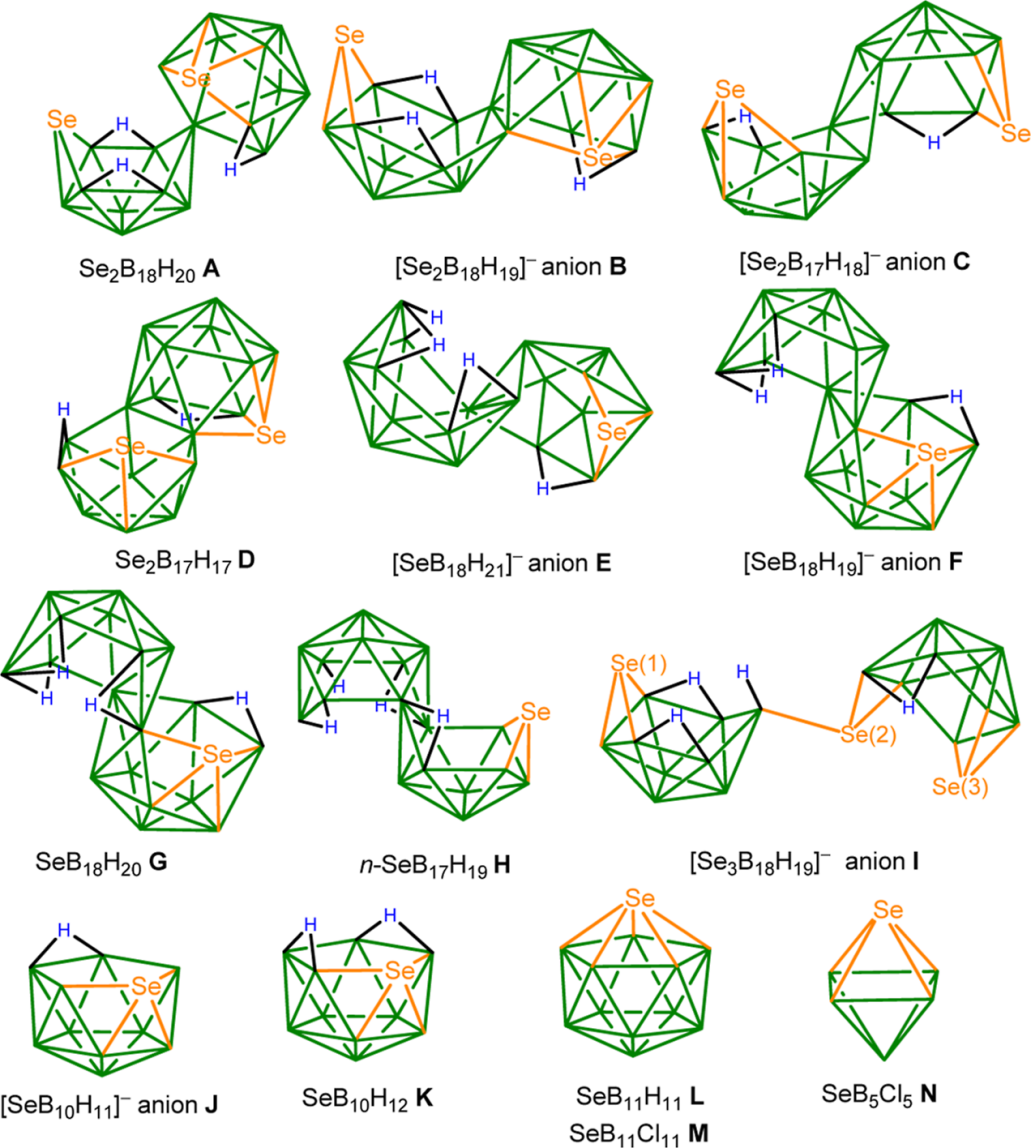
Schematic Structures of the Measured Selenaboranes Listed in Table 2

This paper deals primarily with the comparison of the calculated and measured ^77^Se chemical shifts of the previously reported neutral and anionic selenaborane cluster compounds of known structure, as shown in Chart 1. Nevertheless, we first describe the characterization of a previously unreported new neutral selenaborane Se_2_B_18_H_20_ (Compound **A**). Data for **A** were not included in our recent paper on the luminescent properties of chalcogen-containing macropolyhedral species,^22^ as the low amounts of the compound available were not sufficient for full photophysical investigation. It is, however, useful to demonstrate how a comparison of the measured and calculated Se-77 chemical shifts can be used to support the proposed structure.

**Scheme 1:**
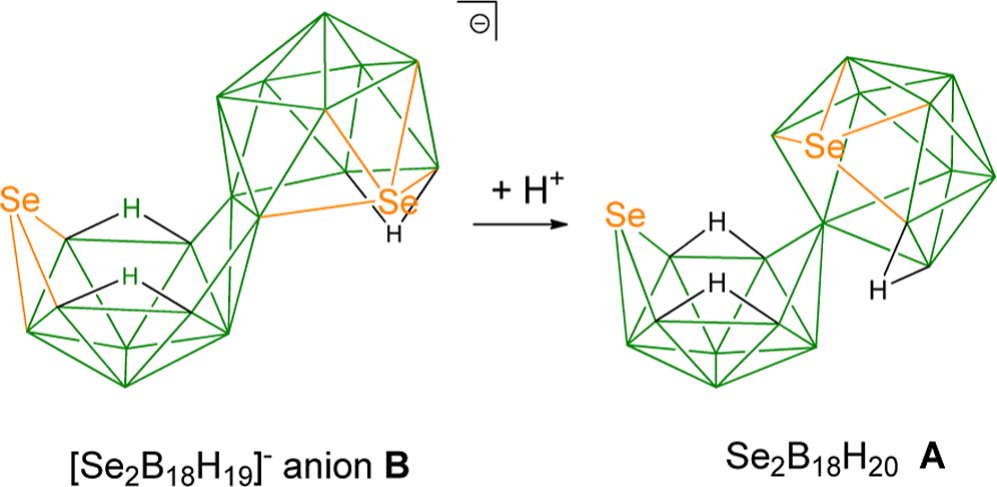
Protonation of [Ph_4_P][Se_2_B_18_H_19_] (**B**) to Afford Se_2_B_18_H_20_ (**A**)

**Figure 1:**
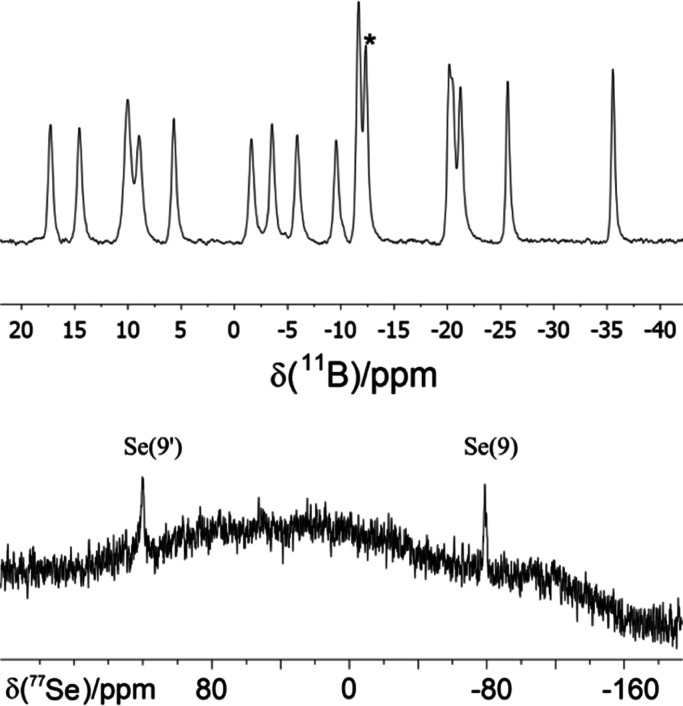
(Upper) ^11^B-{^1^H} NMR spectrum of Se_2_B_18_H_20_, **A**. All resonances are doublets in the ^11^B spectrum except for the singlet resonance due to the *commo*-boron atom linking the two subclusters, as denoted by an asterisk. (Lower) ^77^Se NMR spectrum of **A**.

**Figure 2:**
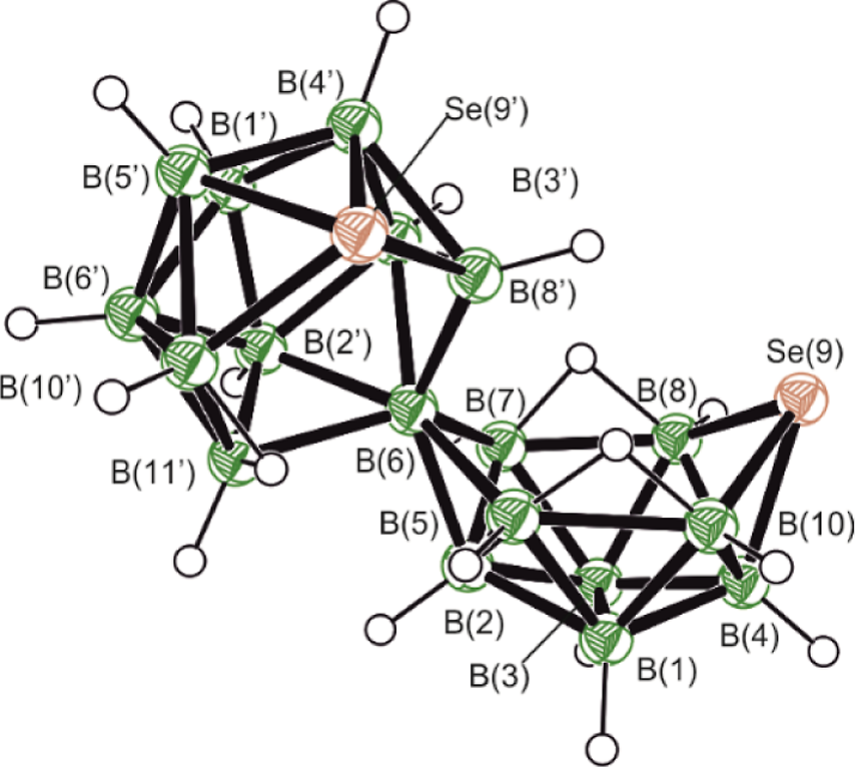
ORTEP-type diagram of the DFT-calculated structure of Se_2_B_18_H_20_**A**, showing the cluster numbering.

Compound **A** is produced almost quantitatively from the protonation of a low-yield (3%) side product, the [Se_2_B_18_H_19_]^−^ anion, formed in the reaction between *syn*-B_18_H_22_ and elemental selenium.^21^ This is in contrast to the higher yield (48%) equivalent reaction between elemental sulfur and *syn*-B_18_H_22_.^39^ Similarly to the reaction for [S_2_B_18_H_19_]^−^, the 20 vertex macropolyhedral species Se_2_B_18_H_20_**A** may be prepared from [Ph_4_P][Se_2_B_18_H_19_] by the addition of H_2_SO_4_ or CF_3_COOH to dichloromethane solutions of the anion (Scheme 1). An almost quantitative formation of protonated compound **A** was observed by boron-11 NMR spectroscopy on acidification of the anion (Figure S1) with H_2_SO_4_. The thiaborane analogue has been described, and its structure deduced from NMR spectroscopy allied with density functional theory (DFT) calculation.^40^ We similarly have only been able to characterize the cluster compound by ^11^B, ^11^B{^1^H}, ^11^B{^1^H selective}, and ^1^H-^11^B HMQC spectroscopy, with the addition of ^77^Se NMR spectroscopy, and by comparison of these with the thiaborane analogue. Our attempts to obtain single crystals of both **A** and the sulfur analogue suitable for X-ray diffraction analysis were unsuccessful. The ^11^B and ^1^H NMR data for **A** are listed in Table 1 together with the boron chemical shift data for S_2_B_18_H_20_, and they show an excellent correspondence between the measured sulfur and selenium species and the calculated boron NMR chemical shifts for **A**. The ^11^B{^1^H} spectrum for **A** and the ^77^Se spectrum are shown in Figure 1. Figure 2 shows an ORTEP-type drawing of the DFT calculated structure together with the cluster numbering. The ^77^Se NMR spectrum in Figure 1 shows two selenium resonances labeled Se(9′) and Se(9). These were assigned by a comparison of the calculated and measured chemical shifts. The means by which we arrived at these assignments are described next.

**Table 1:** Measured Proton and Boron-11 NMR Data for Se_2_B_18_H_20_ (**A**) at 293 K in CDCl_3_ Solution with B3LYP/6-31+G(d,p)/GIAO Calculated Chemical Shifts in Square Brackets and Together with Measured δ(^11^B) NMR Comparison Data for S_2_B_18_H_20_^40^

assign	δ(^11^B)/ppm	δ(^1^H)/ppm	δ(^11^B)/ppm of S_2_B_18_H_20_
B(2′)	+17.3 [+16.7]	+4.27	+15.9
B(2)	+14.6 [+14.4]	+4.91	+15.3
B(4)	+10.1 [+12.6]	+4.19	+9.1
B(5′)	+10.1 [+12.1]	+3.73	+11.1
B(8′)	+9.0 [+11.9]	+3.88	+7.3
B(11′)	+5.7 [+6.3]	+3.46	+5.6
B(4′)	–1.6 [−0.4]	+3.19	–1.9
B(7)	–3.5 [−1.4]	+2.86	–5.1
B(10′)	–5.8 [−5.8]	+2.69	–6.0
B(8)	–9.6 [−6.2]	+2.62	–8.5
B(10) B(1′)	–11.6(2) [−8.5, −11.3]	+3.32, +2.86	–11.6(2)
B(6)	–12.3 [−9.8]	---a	–13.1^a^
B(1)	–20.2 [−19.0]^b^	+2.82	–19.5
B(5)	–20.4 [−18.7]^b^	+1.17	–19.5
B(3′)	–21.2 [−20.6]	+1.57	–22.2
B(6′)	–25.7 [−26.0]	+1.56	–24.2
B(3)	–35.5 [−34.9]	+1.75	–34.9
μH(5,10)		+0.57	+1.00
μH(7,8)		–1.20	–0.88
μH(10′,11′)		–1.62	–1.14

a *Commo*-boron position and therefore no attached proton.

b It may be noted that the ordering of calculated boron chemical shifts is reversed compared to the measured values. The ^1^H–^11^B HMQC spectrum (Figure S2) shows that the bridging hydrogen resonance at +0.57 ppm is coupled to the boron resonance at −20.4 ppm as would be expected if the boron resonance is due to vertex B(5). Some cross peaks are not found, but these were located by selectively decoupled boron in the proton spectra, ^1^H{^11^B_selective_}, as shown in Figure S3. Additionally, as we have noted before,^41^ it is often possible to differentiate the assignments of close boron resonances from calculation by also looking at the calculated values for their individual directly attached *exo*-terminal proton resonances. Thus, for B(5)-H, the calculated value is +1.17 ppm compared to that for B(1)-H, where it is +2.93 ppm. These nicely match the measured values from the HMQC spectrum of +1.17 and +2.82 ppm, respectively, thereby supporting the assignments given in the table.

### Calculations

**Figure 3:**
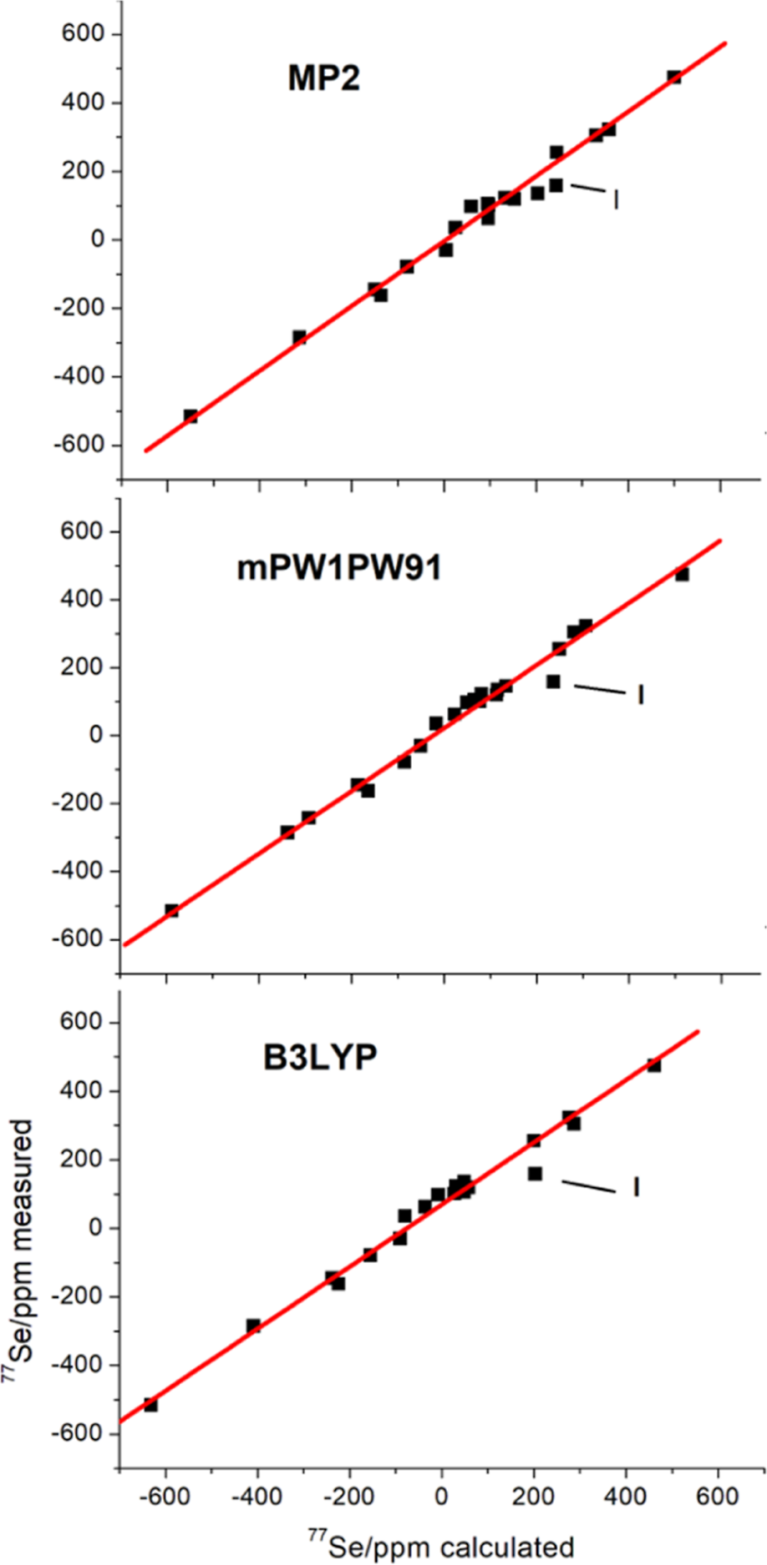
Plots of measured versus calculated δ(^77^Se) chemical shifts at the DFT/B3LYP, DFT/mPW1PW91-GIAO, and MP2 levels. The lines represent the linear regression analyses δ_calc_ = *A* × δ_exp_ + *B* with B3LYP, *A* = 0.886, *B* = 66, *R*^2^ = 0.983; mPW1PW91, *A* = 0.912, *B* = 23, *R*^2^ = 0.988; MP2, *A* = 0.925, *B* = −9, *R*^2^ = 0.986. The data point labeled **I** is the most prominent outlier point. Numerical data are listed in Tables S1–S3.

Precisely calculated values for heavy elements such as ^77^Se may ideally include relativistic corrections using routines other than DFT methods, but these would require considerable computing power for even simple selenium-containing compounds.^36^ GIAO-MP2 methods have been used to good effect in the calculation of ^77^Se chemical shieldings.^34^ These, however, are also computationally expensive, and we therefore compare them to less expensive DFT methods that may be more routinely used in the characterization of selenaborane compounds. Here, we parallel lower-level DFT calculated isotropic shielding constants using B3LYP and mPW1PW91 functionals to those of MP2 (2nd order Møller–Plesset perturbation theory). In all cases, use the same 6-31+G(d,p) basis set for the main group elements B, H, and Cl and with the Binning and Curtiss 962 + d polarization basis set for Se, as used previously by Buehl et al.^34^ The calculated chemical shift values presented in Table 2 are derived from linear regression analyses of the calculated isotropic shielding relative to the calculated value for the dimethylselenide reference at the appropriate level versus the measured chemical shifts. Plots of the data are shown in Figure 3 together with the linear regression parameters. The three methods can be seen to produce very similar values for *R*^2^, the coefficient of determination, and the two relatively low-cost DFT methods, which are not significantly different from the more computationally costly MP2 calculations. The mPW1PW91/6-31+G(d,p) method shows a good correlation between measured and calculated values, and these will be used in further discussions.

**Table 2:** Measured and Calculated ^77^Se NMR Chemical Shifts in CDCl_3_

			4/5-connected^b^				3-connected^c^			
ref	compound^a^		meas	B3LYP^d^	mPW1PW91	MP2^d^	meas	B3LYP^d^	mPW1PW91	MP2^d^
this work^e^	**A**	Se_2_B_18_H_20_	+119	+118	+127	+138	–79	–71	–55	–77
(21)	**B**	[Se_2_B_18_H_19_]	+62	+35	+44	+86	–516	–493	–514	–508
(21)	**C**	[Se_2_B_17_H_18_]					+35	–4	+8	+20
(22)	**D**	Se_2_B_17_H_17_	+135	+110	+129	+185	+122	+94	+97	+119
(21)	**E**	[SeB_18_H_21_]					–163	–132	–126	–128
(21)	**F**	[SeB_18_H_19_]	+304	+321	+280	+301				
(22)	**G**	SeB_18_H_20_	+322	+312	+303	+326				
(22)	**H**	SeB_17_H_19_					+474	+476	+493	+457
(20)	**I**	[Se_3_B_18_H_21_]					–286	–295^f^	–285^f^	–290^f^
							+97	+60^g^	+63^g^	+52^g^
							+158	+247^h^	+239^h^	+221^h^
this work^e^	**J**	[SeB_10_H_11_]	+105	+109	+82	+85				
this work^e^	**K**	SeB_10_H_12_	+100	+91	+93	+86				
this work^e^	**L**	SeB_11_H_11_	+254^i^	+244	+251	+223				
(31)	**M**	SeB_11_Cl_11_	–31^i^	–13	–23	+1				
(31)	**N**	SeB_5_Cl_5_	–146	–144	–147	–140				

a Square brackets indicate that the compound is monoanionic. See Chart 1 for schematic structures of the compounds and Figures 1 and S4–S16 for the^77^Se measured spectra.

b 4-Connected selenium on an 11-vertex subcluster.

c 3-Connected selenium on a 10-vertex subcluster.

d Chemical shift values estimated from the linear regression analysis shown below in Figure 3.

e The [SeB_10_H_11_]^−^ anion^25^ was obtained as a byproduct in the synthesis of [Se_2_B_17_H_18_]^−^,^21^ and its conjugate acid, SeB_10_H_12_, was obtained by acidification of the anion with H_2_SO_4_. SeB_11_H_11_ was donated by Josef Holub.^42^

f Se(1), see Chart 1.

g Se(2), see Chart 1.

h Se(3), see Chart 1.

i 5-Connected selenium.

Although there is no way to definitely assign the calculated values to the measured values in the multiselenium compounds, the close conformity of the calculated and measured values in the compounds containing a single selenium vertex, including the perchlorinated species SeB_11_Cl_11_**M** and SeB_5_Cl_5_**N**, allows us a large degree of confidence in the assignments in those macropolyhedral species where the identities of the measured selenium resonances cannot be directly assigned. The differences in the chemical shifts between the sites in the macropolyhedral species are, in most cases, considerably greater than the standard deviations in the calculated values. This is, however, currently a limited set of data.

In the three methods, there is one prominent outlier point (**I** in Figure 3). This arises from the [(Se_2_B_9_H_10_)(SeB_9_H_11_)]^–^ anion, compound **I** in Chart 1. Here, the 3-connected Se(3), vertex held on a *nido* cluster, affords a measured value of δ(^77^Se) + 158 ppm and a calculated value of +239 ppm, making it the selenium atom presenting the largest deviation for all three methods. In contrast, the Se(1) held on an *arachno* cluster in the same molecule gives δ(^77^Se) −286 ppm, which is very close to the calculated value of −285 ppm, and also a bridging Se(2) center with very close measured and calculated chemical shifts of +97 and +63 ppm, respectively. This is an indication of the large effect that the local cluster environment can have on the shielding around the selenium centers and that calculated values may potentially vary quite widely, even in the same molecule. The two clusters in [(Se_2_B_9_H_10_)(SeB_9_H_11_)]^−^ are linked by a two-electron, two-center single bond, suggesting that there can be free rotation around the linkage. This molecular flexibility may introduce effects not well modeled by the calculated static structures. Nevertheless, in nearly all cases, each calculational method produces the chemical shifts in the same order as the measured values. Thus, the ordering of the higher and lower field resonances in the measured macropolyhedral selenaboranes is always mirrored by the calculations. This is also true for compound **I** above. The only exception is in the 11-vertex *nido* clusters for the mPW1PW91/6-31+G(d,p) level, where the ordering of the very close measured values for the anion [SeB_10_H_11_]^−^**J** and SeB_10_H_12_**K** at +105 and +100 ppm is reversed in the calculated chemical shifts (+82 and +93 ppm, respectively). This indicates that the predictions for separate molecules with very close chemical shifts are not reliable. Nevertheless, the predictions are still sufficiently close to the observed chemical shifts to allow the resonances to be located easily during the measurement.

Overall, with this small sample of compounds, the anionic species seem to appear generally at higher field than the neutral species as shown in the **A**/**B**, **G**/**F**, and **K**/**J** conjugate acid/base pairs.

**Figure 4:**
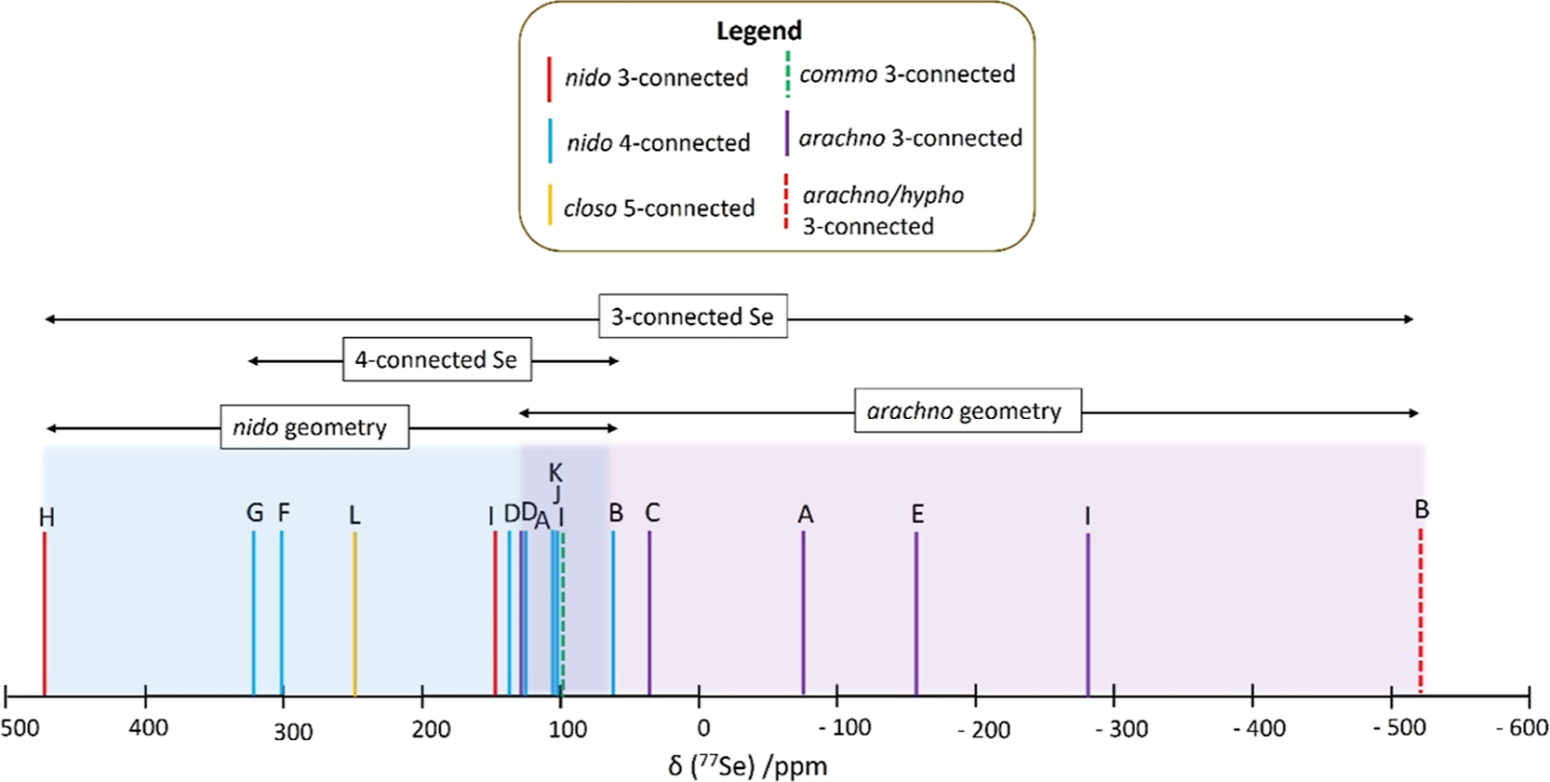
Schematic illustration of the distribution of ^77^Se resonances in selenaborane species. See Chart 1 for compound identity.

Figure 4 illustrates the distribution of the selenium resonances in polyhedral boron hydride species **A** to **L**. The perchlorinated *closo* species **M** and **N** are not considered here, as they constitute a separate subgroup. From this presentation of the data, two trends are revealed. First, the measured ^77^Se chemical shifts in the *nido* subclusters with a 4-connected Se vertex span a smaller range (from +62 to +322 ppm) compared to the subclusters with a 3-connected Se vertex, which span a much larger range of almost a 1000 ppm (from +474 ppm in neutral SeB_17_H_19_, **H**, to −516 ppm in anionic [Se_2_B_18_H_19_]^−^**B**). Second, the data so far suggest that selenium vertices on the *nido* clusters and subclusters are to lower field than that for the *arachno* subclusters.

**Chart 2:**
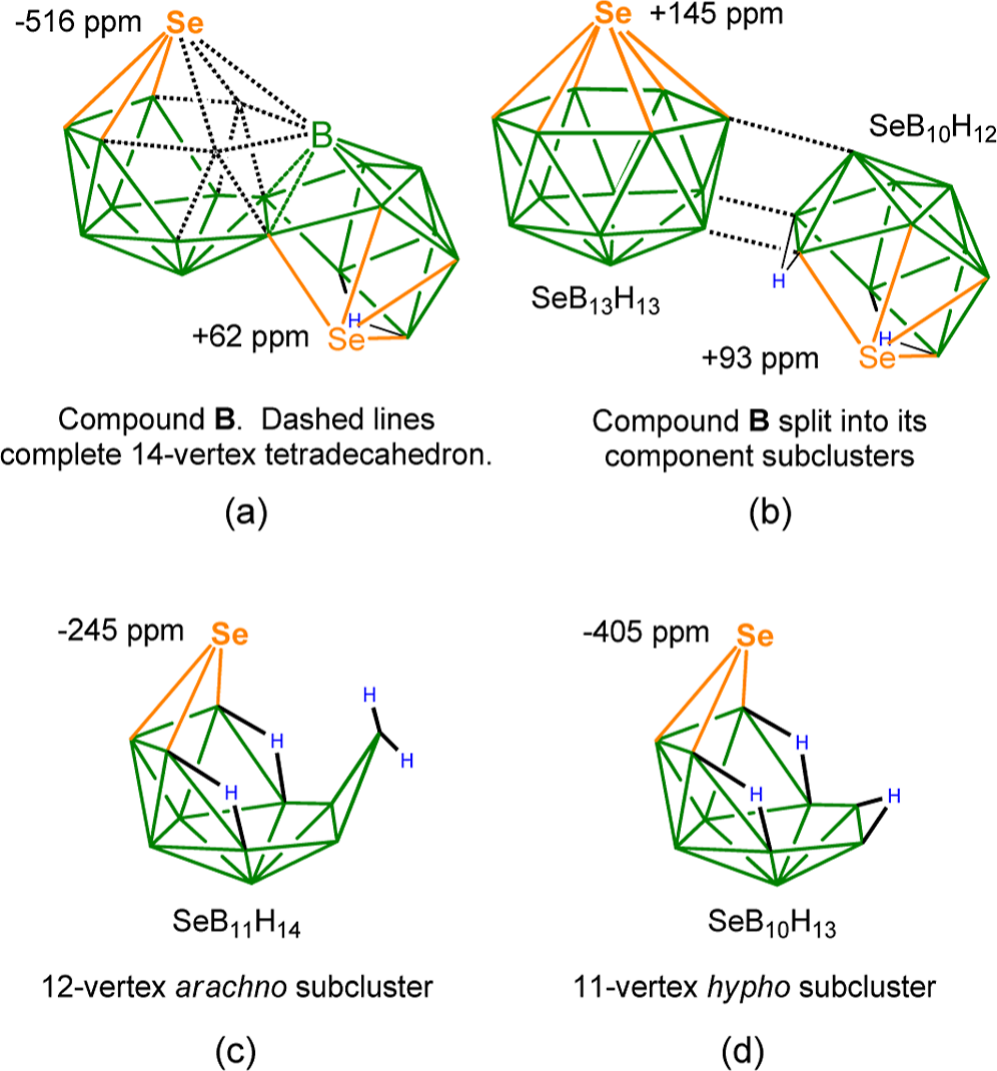
Structural Considerations of the [Se_2_B_18_H_19_]^−^ Anion (B). (a) Measured Chemical Shifts and (b–d) Calculated Chemical Shifts at the mPW1PW91 Level.

Also noteworthy is the resonance in the 20-vertex [Se_2_B_18_H_19_]^−^ anion **B** at −516 ppm, which is 230 ppm more shielded than the closest next resonance—the *arachno* 3-connected Se(1) vertex in **I** (−286 ppm). The extent of this difference led us to consider its reason. Thus, in **B**, one subcluster contains a rare 6-connected boron vertex,^21,43,44^ and the subcluster could be regarded as being based geometrically on a *closo*-14-vertex tetradecahedron^45,46^ of either *arachno* 12-vertex constitution with 2 missing vertices or of *hypho* 11-vertex geometry based on 3 missing vertices (Chart 2a) depending on whether or not the boron atom labeled B is included in or excluded from the subcluster vertex count. In both of these, the selenium vertex is in an antipodal position with respect to the 6-connected boron in the tetradecahedron. We consequently hypothesized that the chemical shift might be due to the antipodal effect^47^ of the boron vertex, and we therefore calculated the chemical shift of selenium in a hypothetical *closo*-SeB_13_H_13_ cluster using the mPW1PW91 level regression analysis. However, as shown in Chart 2b, the chemical shift of +145 ppm is in the same region as for *closo*-SeB_11_H_11_ (**L**) and no extreme effect of the unusual boron vertex is evident. Interestingly, the chemical shift of the 4-connected selenium vertex directly adjacent to the boron atom in the *dicommo* linkage that is protonated on the acidification of [Se_2_B_18_H_19_]^−^ (see Scheme 1) does not undergo a large chemical shift change (+62 to +119 ppm), whereas the more remote 3-connected selenium changes from −79 to −516 ppm. This indicates that the second subcluster has a large influence on the electronic environment of the selenium. Separate calculations of the δ(^77^Se) for the hypothetical *arachno* and *hypho* subcluster geometries, Chart 2c,d, respectively, show a much better correlation between the 11-vertex *hypho* constitution and compound **B** (see Chart 2), suggesting this to be the better description of its subcluster geometry and may account for its increased shielding.

Finally, as we noted earlier in the description of the characterization of Se_2_B_18_H_20_**A**, primarily by ^11^B and ^1^H NMR spectroscopy and their comparison to those of S_2_B_18_H_20_, the prediction of the selenium chemical shifts by all three methods gives very close correlations to the measured values, and they therefore give independent confirmation of the proposed structures of both Se_2_B_18_H_20_ and S_2_B_18_H_20_.

## Conclusions

This work indicates that fast and computationally inexpensive lower-level quantum chemical calculations are sufficiently accurate to enable the assignment of measured data in macropolyhedral selenaborane species. There are, however, currently only a limited number of examples from which to make this assessment, and we hope to expand this data set with further work. Nevertheless, being able to predict ^77^Se NMR chemical shifts in selenaborane cluster compounds offers several benefits.

1 The chemical shift of the selenium nucleus in a selenaborane cluster compound can provide information about its molecular structure. Accurate prediction of these chemical shifts allows researchers to better understand the arrangement of atoms within the cluster, as well as subcluster geometries (*nido*, *arachno*, etc.).
2 Accurately predicted chemical shifts enable the position of ^77^Se resonances to be more efficiently located in experimental measurements. Indeed, in the final sample we measured, SeB_18_H_20_, for which only a small amount of material was available, requiring a long accumulation time, we looked in the predicted region of zero to +400 ppm and found the measured resonance at +322 ppm (calculated: +303 ppm).
3 Reliable prediction of ^77^Se NMR chemical shifts aids the interpretation of experimental spectra, permitting the assignment of specific signals to different structural motifs within selenaborane cluster compounds. This may aid in the identification and characterization of these compounds in complex mixtures or reaction intermediates.

## Experimental Section

### Caution

Although **s**elenium is a biologically useful trace element, it is toxic,^48^ and selenium-containing compounds should be handled accordingly.

### NMR Spectroscopy

NMR spectra were recorded on a JEOL ECZ 600 R (14.1 T) spectrometer using ^77^Se, ^11^B, ^11^B{^1^H}, ^1^H, ^1^H{^11^B(broadband)}, ^1^H{^11^B(selective)}, and HMQC (heteronuclear multiple-quantum correlation) techniques. NMR spectra of all compounds were measured in a CDCl_3_ solution. ^11^B chemical shifts given relative to BF_3_·OEt_2_, δ^11^B = 0.0 ppm for Ξ (^11^B) = 32,083,971 Hz. ^77^Se chemical shifts are reported relative to SeMe_2_, δ(^77^Se) = 0.0 ppm for Ξ (^77^Se) = 19,071,523 Hz.

The ^77^Se NMR spectra were measured using a standard single-pulse sequence (available from the spectrometer library) with a 90° pulse length and relaxation delay of 0.1–0.2 s. The line widths of the ^77^Se resonances were in the range of 200–300 Hz, and the spectra were recorded with an FID resolution of 3–4 Hz. Due to the absence of the hydrogen atoms attached to the Se atom, the spectra were acquired without proton decoupling.

### Computational Details

Calculations were performed using Gaussian16 package.^49^ For the DFT/B3LYP methodology, the 6-31+G(d,p) basis sets for B, Cl, and H were used, and the Binning and Curtiss 962 + d polarization basis set for Se was taken from Basis Set Exchange.^50^ The higher level MP2 calculations were carried out using the same basis sets. The polarizable continuum model was implemented with CHCl_3_ solvation. Frequency analyses to confirm the true minima were performed at the appropriate level.

### Preparation of Se_2_B_18_H_20_ (**A**)

The compound was prepared by adding excess concentrated H_2_SO_4_ to a CH_2_Cl_2_ solution of [Ph_4_P][Se_2_B_18_H_19_] in a small sample tube. The mixture was shaken, then allowed to settle, and the upper CH_2_Cl_2_ layer was decanted. The solvent was removed under a stream of nitrogen and redissolved in CDCl_3_ for NMR spectroscopic measurement.

## Supporting Information Available

Boron and proton NMR spectra for compound **A**, plots of ^77^Se NMR spectra for those compounds measured by the authors, tables of calculated chemical shieldings versus measured chemical shifts, and tables of calculated Cartesian atomic coordinates (PDF)
