# Supplementary material for: Experimental and Computational 77Se NMR Spectroscopic Study on Selenaborane Cluster Compounds
Source: Inorg Chem. 2024 Aug 20;63(35):16186–93. doi: 10.1021/acs.inorgchem.4c01890 (PMC11372749; doi:10.1021/acs.inorgchem.4c01890)
Supplement: Supplementary file 1 — ic4c01890_si_001.pdf [file ic4c01890_si_001.pdf]

Supporting Information.

Experimental and Computational ^{77}Se NMR Spectroscopic Study on Selenaborane Cluster Compounds

Jonathan Bould,* Michael G. S. Londesborough and Oleg L. Tok.

Institute of Inorganic Chemistry of the Czech Academy of Sciences
Husinec-Řež 250 68, Czech Republic.

Email: bould@iic.cas.cz

CONTENTS

Figure S1	Boron-11 NMR of $CDCl_3$ reaction solution of $Se_2B_{18}H_{20}$ (**A**) after the addition of H_2SO_4 to $[Ph_4P][Se_2B_{18}H_{19}]$.	1
Figure S2	^1H–^{11}B HMQC spectrum for $Se_2B_{18}H_{20}$	2
Figure S3	Selective ^1H-$\{^{11}B_{selective}\}$ spectra for compound **A**.	3
Figures S4 to S16	Measured ^{77}Se NMR spectra for compounds **A** to **N**.	4-10
Table S1	Data for plot of measured and DFT calculated chemical shieldings	11
Table S2	Data for plot of measured and mPW1PW91 calculated chemical shieldings	12
Table S3	Data for plot of measured and MP2 calculated chemical shieldings	13
Tables S4 to S17	MP2 calculated Cartesian coordinates for compounds **A** to **N**.	14
Tables S18 to S31	B3LYP calculated Cartesian coordinates for compounds **A** to **N**.	26
Tables S32 to S45	mPW1PW91 calculated Cartesian coordinates for compounds **A** to **N**.	38

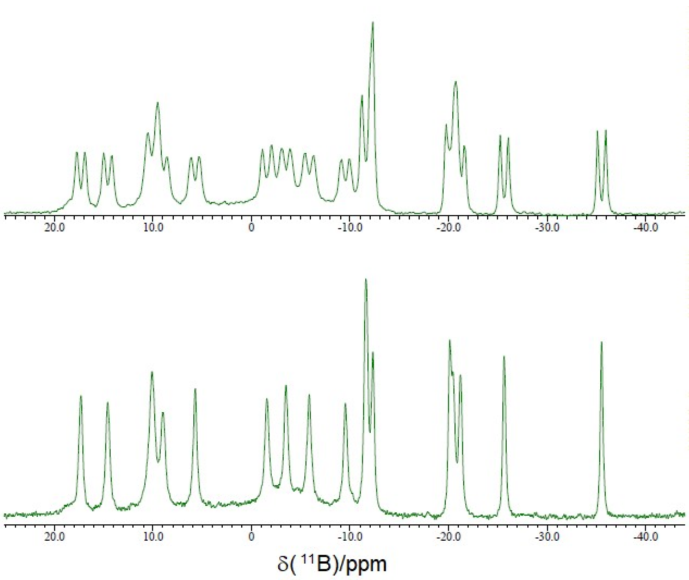

Figure S1. Boron-11 NMR of a $CDCl_3$ reaction solution of $Se_2B_{18}H_{20}$ (**A**) formed after the addition of H_2SO_4 to $[Ph_4P][Se_2B_{18}H_{19}]$.

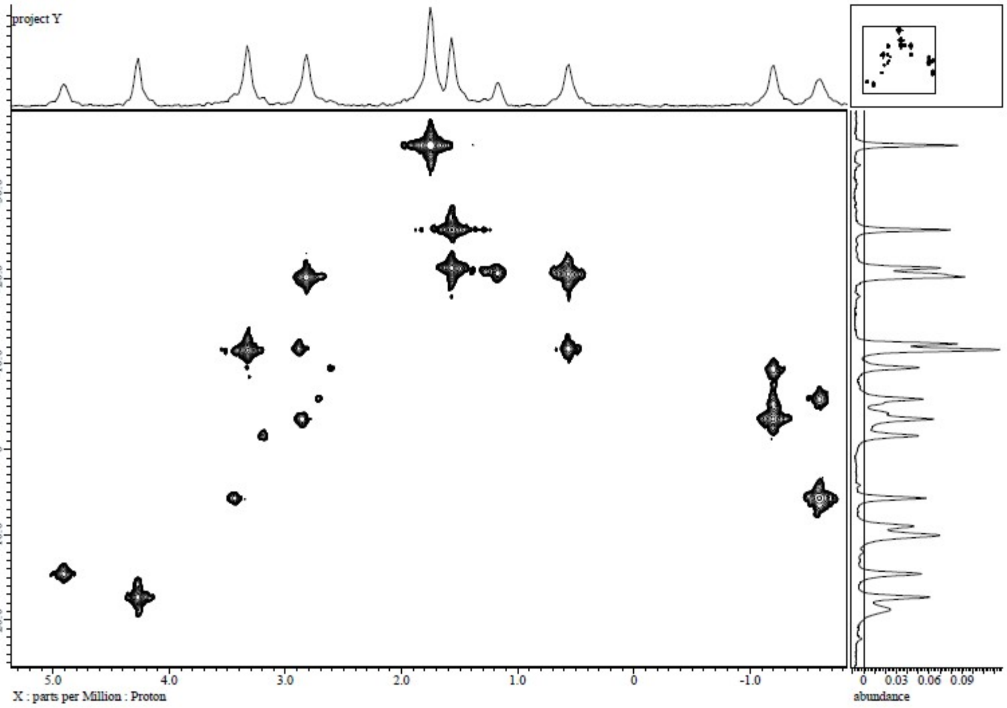

Figure S2. ^{1}H-^{11}B HMQC spectrum for $Se_2B_{18}H_{20}$, **A**, in $CDCl_3$, 291 K. Some cross peaks are not found but these are located in the selectively decoupled proton spectra, ^{1}H-$\{^{11}B_{selective}\}$, shown in Figure S4.

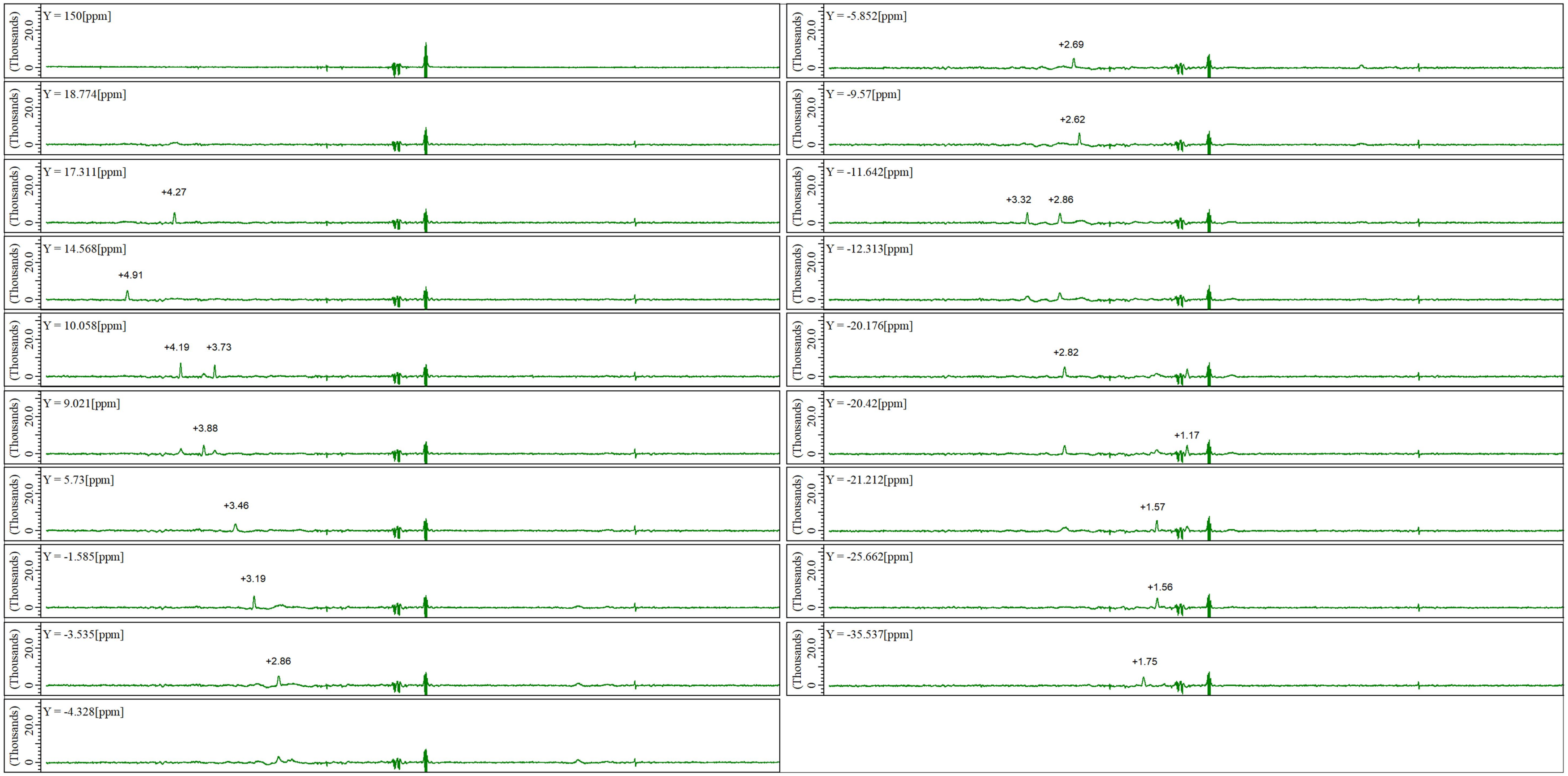

Figure S3. ^{1}H-{$^{11}B_{selective}$} spectra for compound **A**. Spectra are obtained by subtracting the off-resonance (+150 ppm) ^{1}H-{^{11}B} spectrum from the on-resonance spectrum for each boron resonance in compound **A**. This eliminates those proton resonances not coupled to the individual boron resonance. This technique is more suitable for *exo*-terminal proton resonances. HMQC spectra, Figure S3, are more suitable for bridging protons.

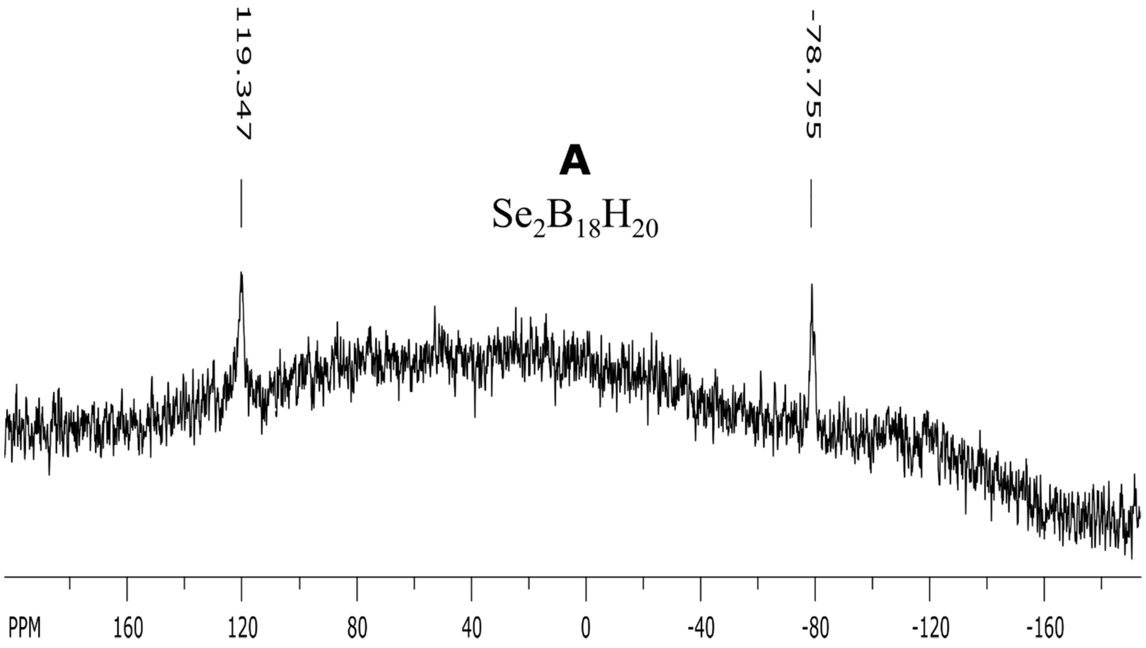

Figure S4. ^{77}Se NMR spectrum (114.46 MHz, $CDCl_3$) of compound **A**.

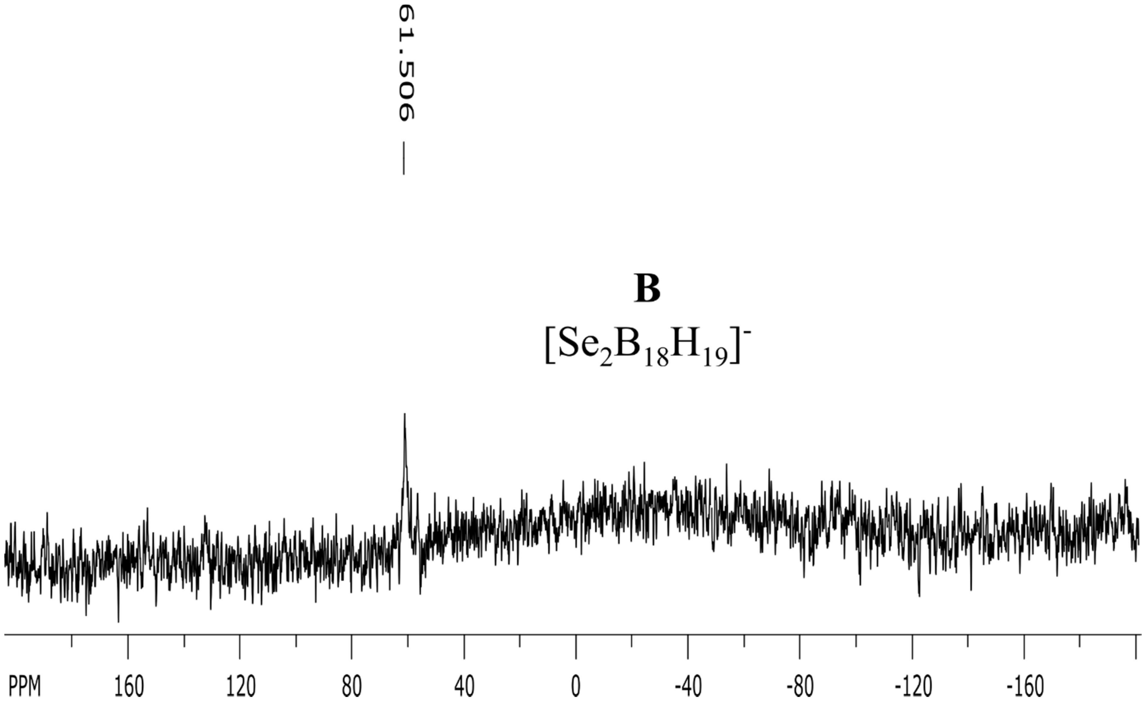

Figure S5. ^{77}Se NMR spectrum (114.46 MHz, $CDCl_3$) of compound **B** (low-field region).

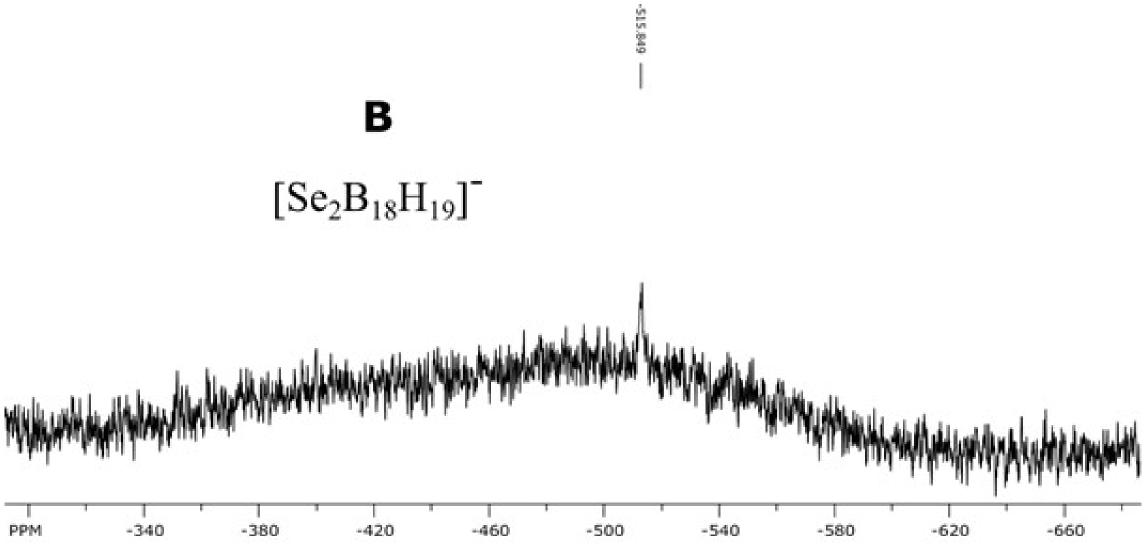

Figure S6. ^{77}Se NMR spectrum (114.46 MHz, $CDCl_3$) of compound **B** (high-field region).

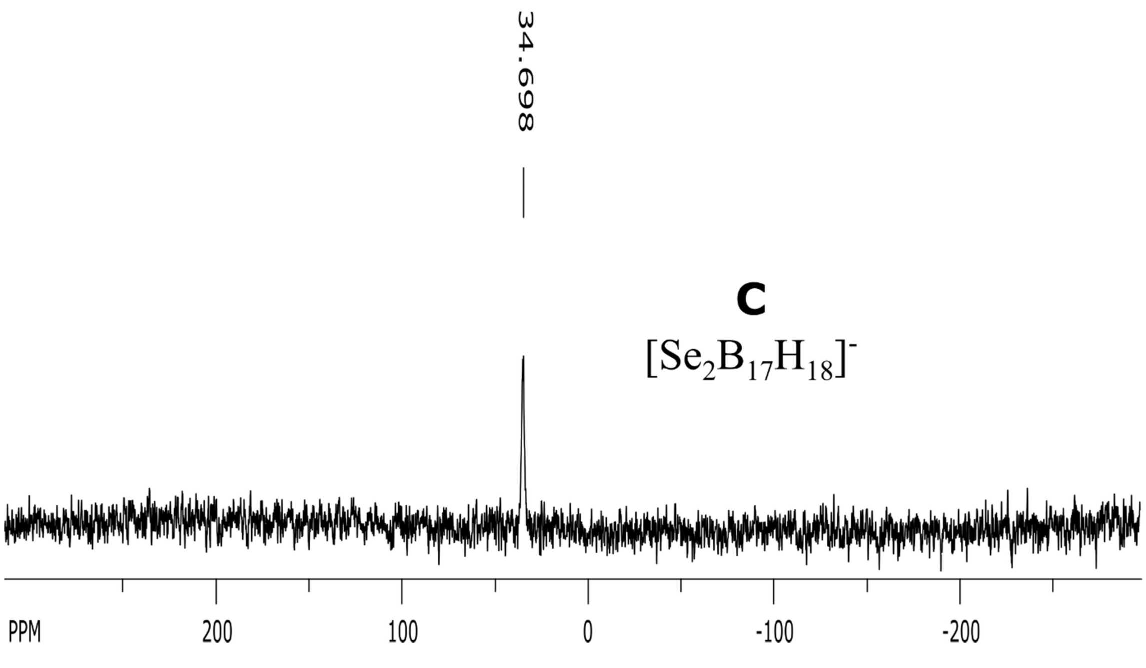

Figure S7. ^{77}Se NMR spectrum (114.46 MHz, $CDCl_3$) of compound **C**.

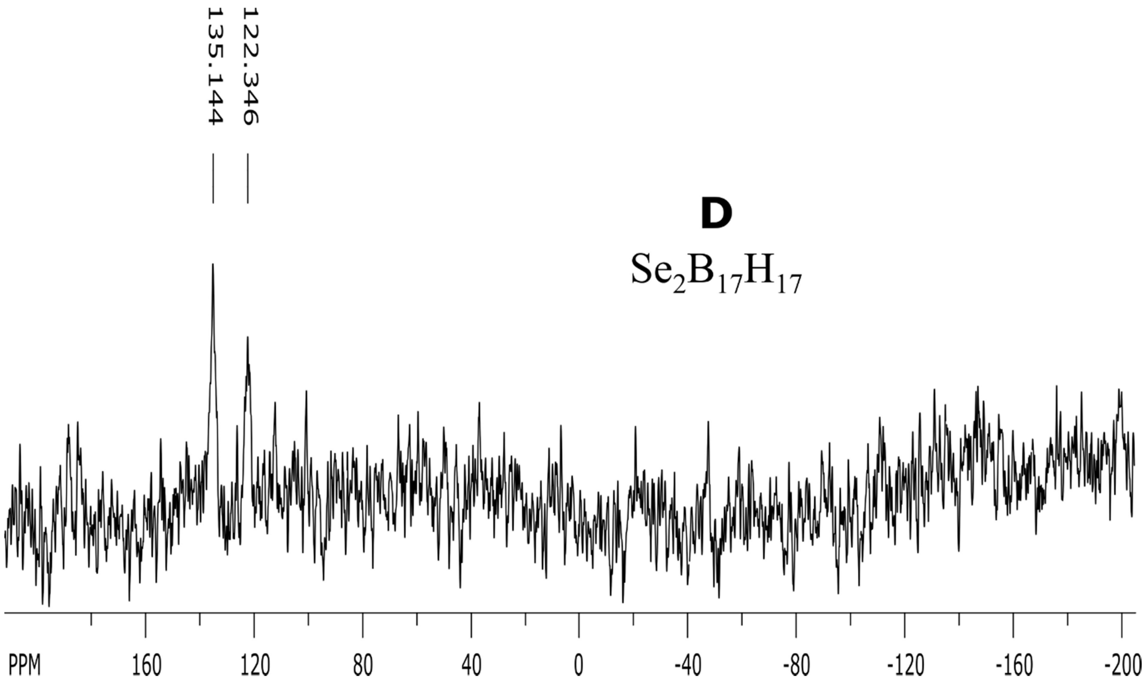

Figure S8. ^{77}Se NMR spectrum (114.46 MHz, $CDCl_3$) of compound **D**.

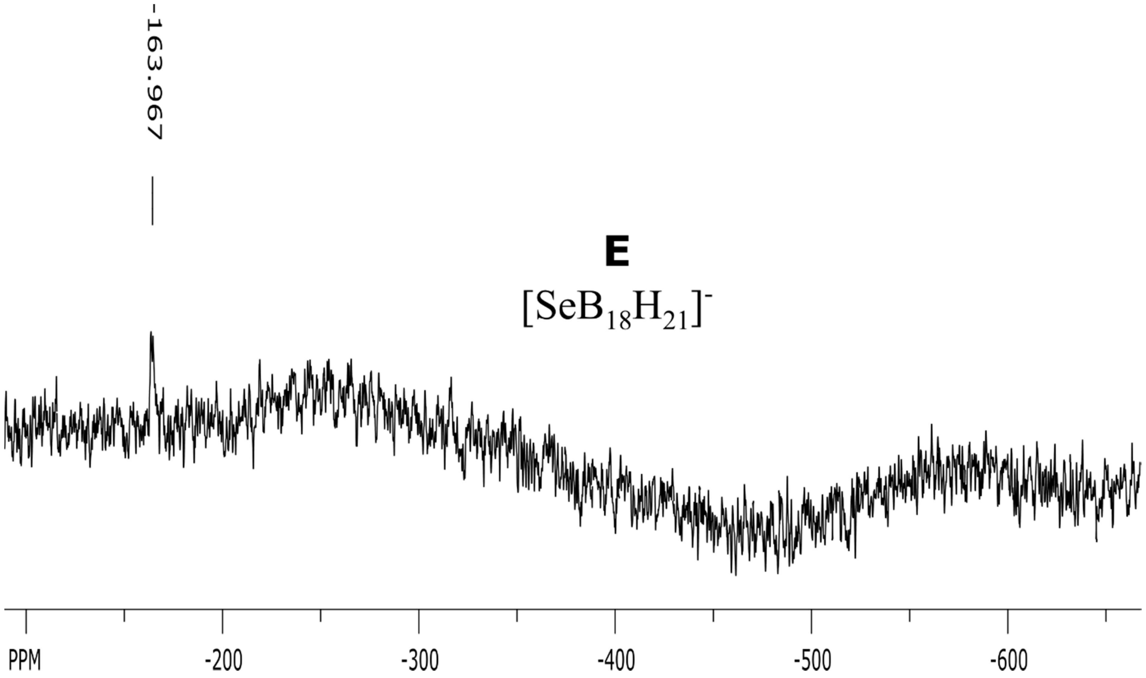

Figure S9. ^{77}Se NMR spectrum (114.46 MHz, $CDCl_3$) of compound **E**.

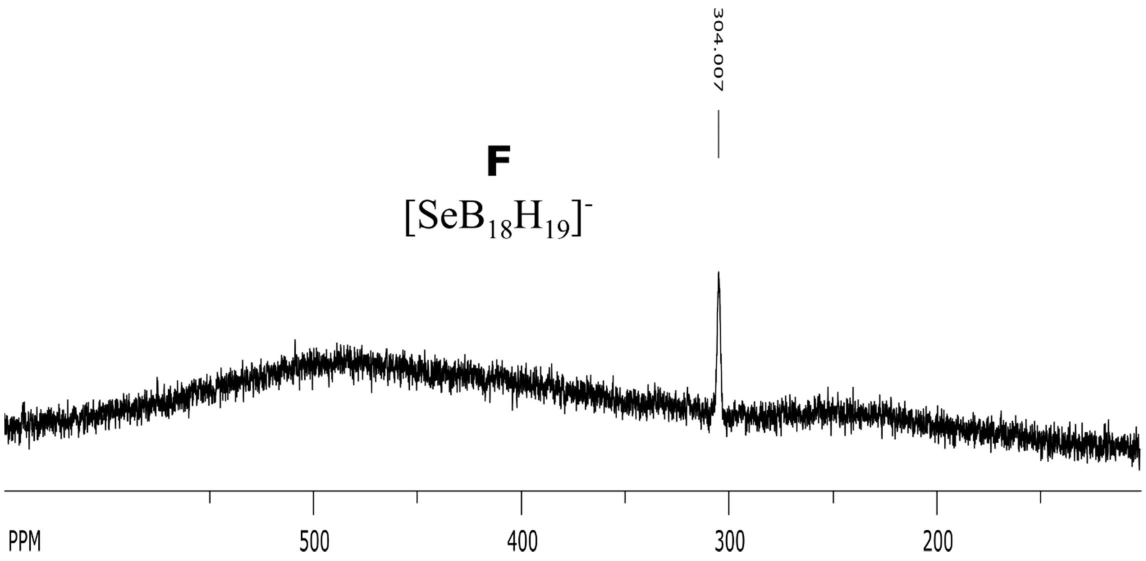

Figure S10. ^{77}Se NMR spectrum (114.46 MHz, $CDCl_3$) of compound **F**.

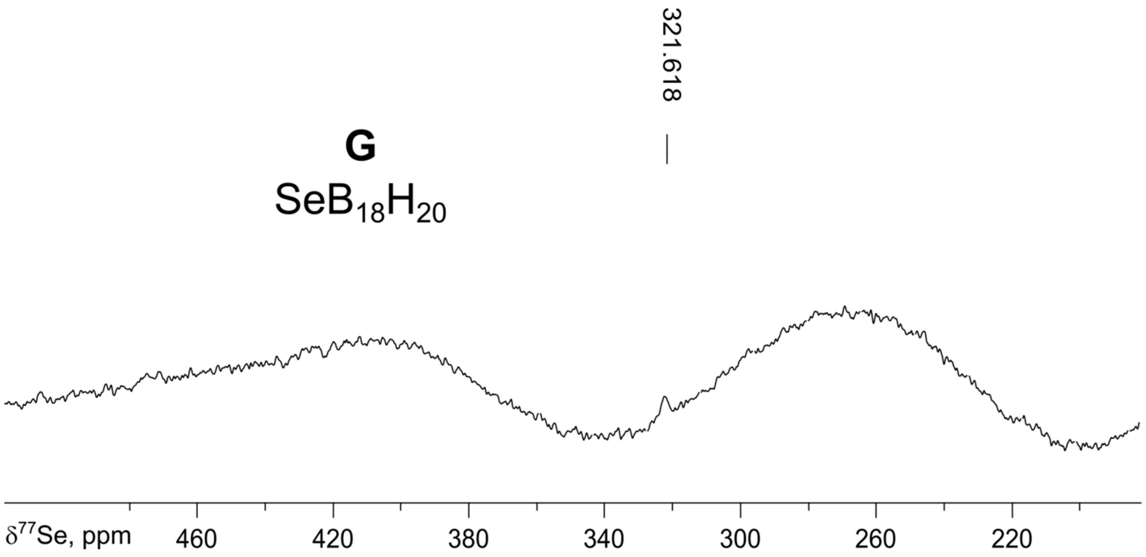

Figure S11. ^{77}Se NMR spectrum (114.46 MHz, $CDCl_3$) of compound **G**.

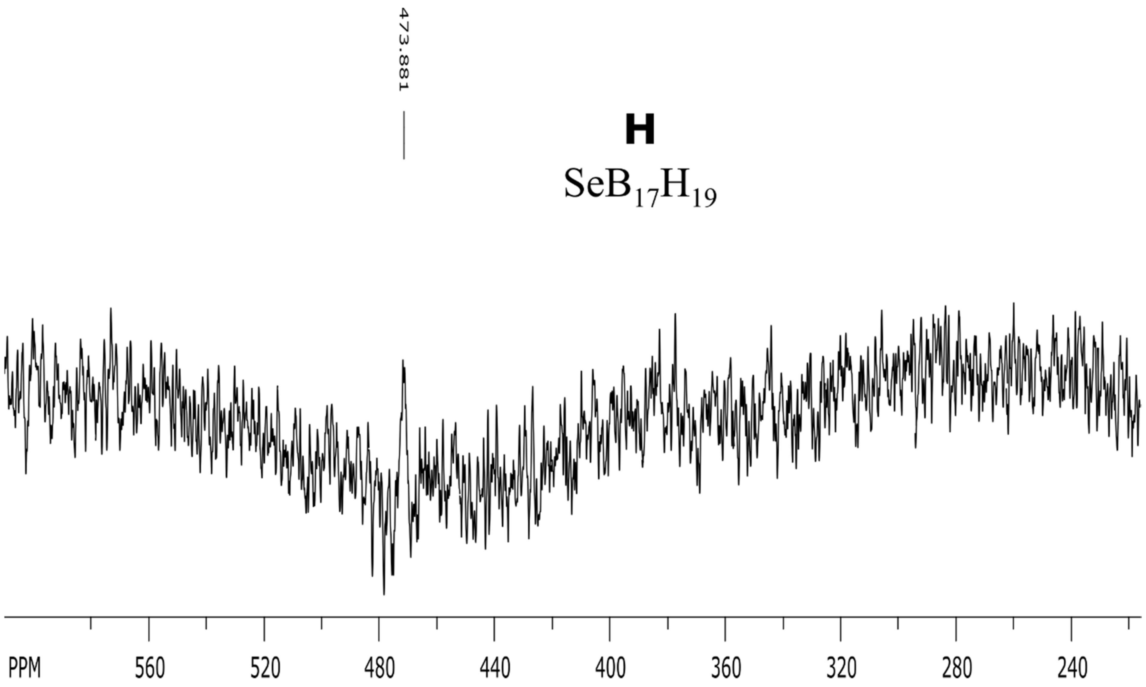

Figure S12. ^{77}Se NMR spectrum (114.46 MHz, $CDCl_3$) of compound **H**.

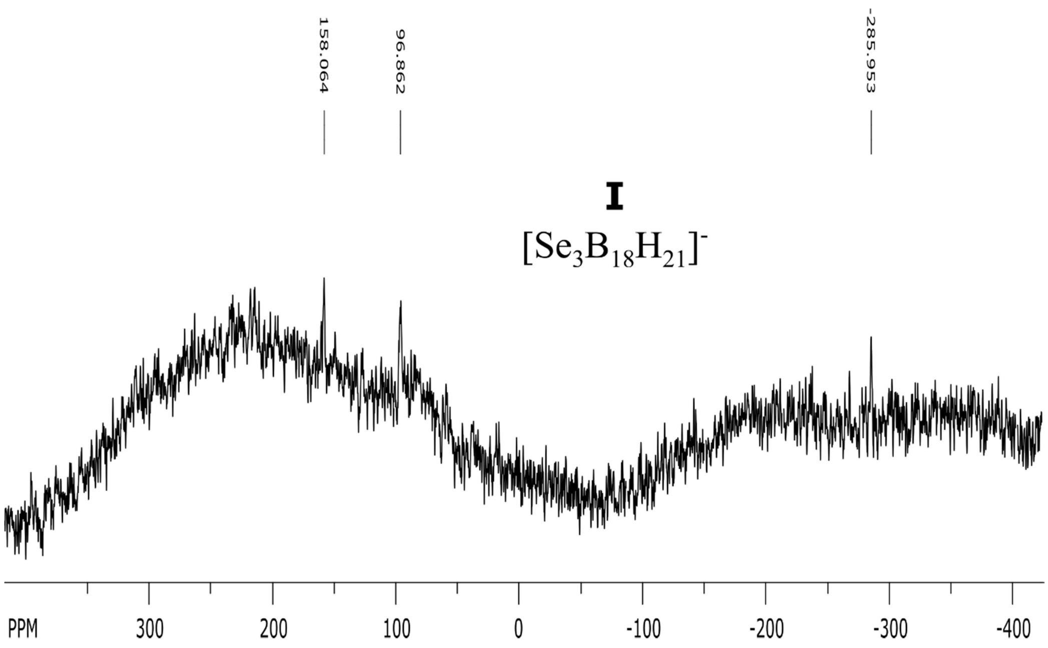

Figure S13. ^{77}Se NMR spectrum (114.46 MHz, $CDCl_3$) of compound **I**.

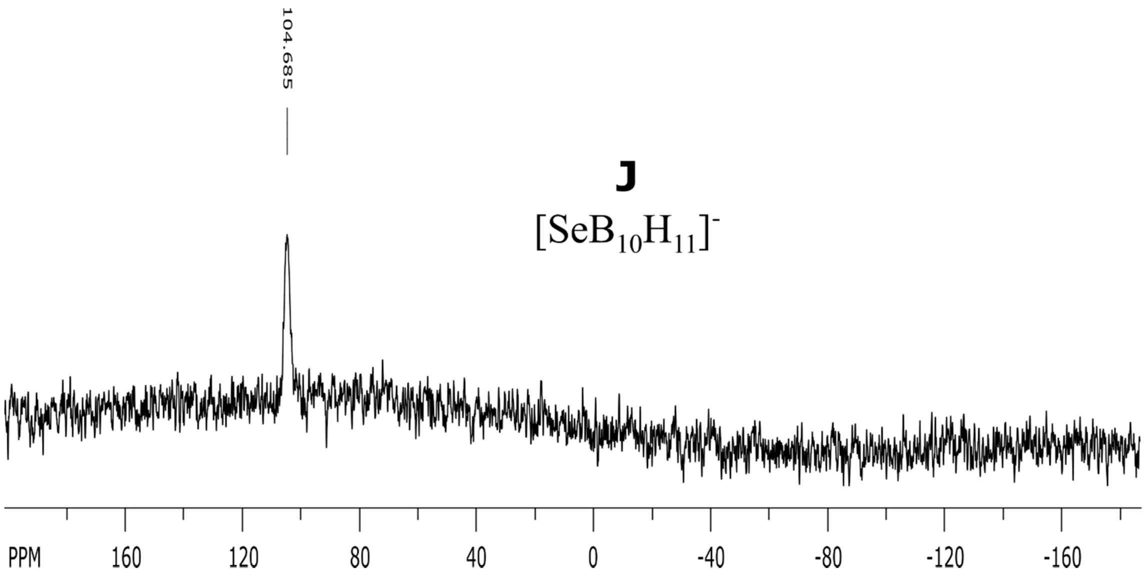

Figure S14. ^{77}Se NMR spectrum (114.46 MHz, $CDCl_3$) of compound **J**.

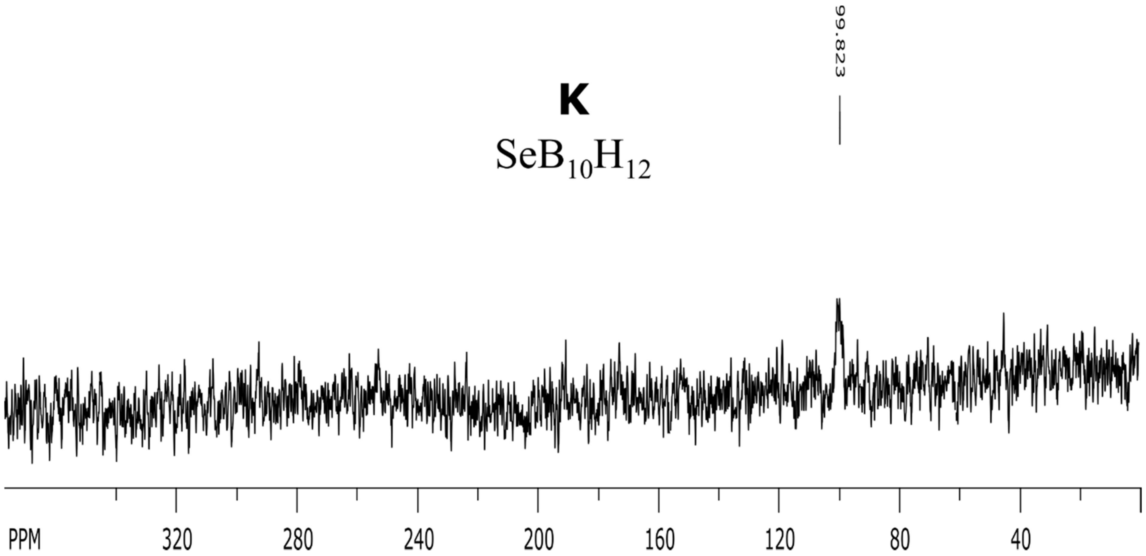

Figure S15. ^{77}Se NMR spectrum (114.46 MHz, $CDCl_3$) of compound **K**.

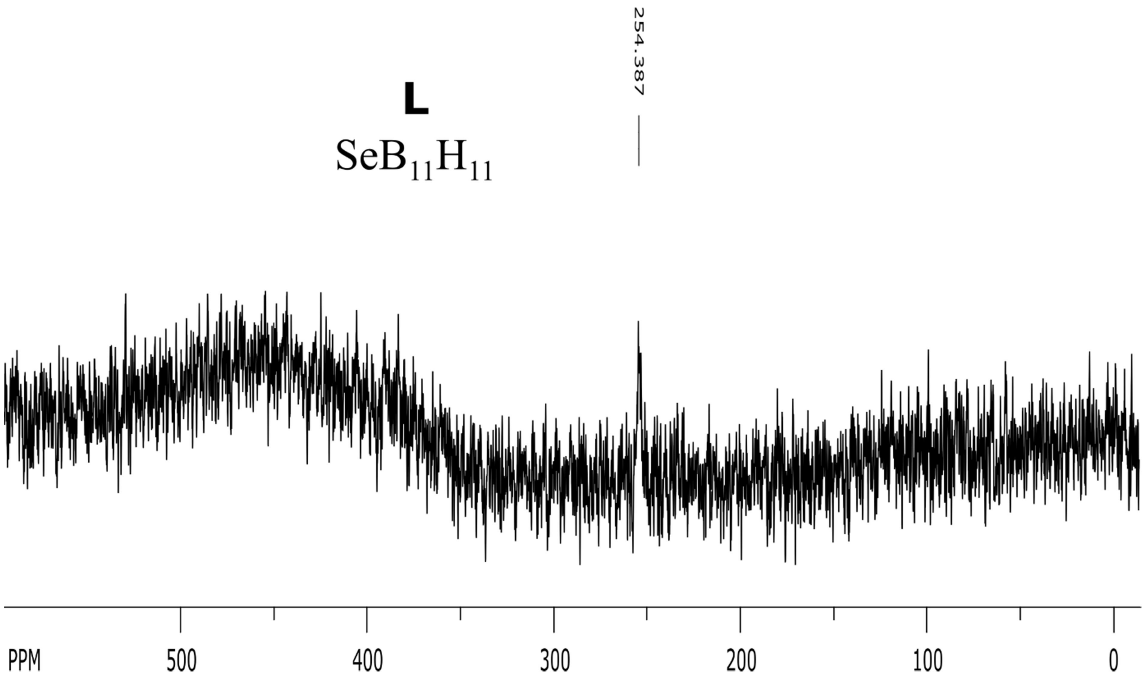

Figure S16. ^{77}Se NMR spectrum (114.46 MHz, $CDCl_3$) of compound **L**.

Table S1. B3LYP/6-31+G(d,p) calculated isotropic chemical shieldings *v.* measured $\delta(^{77}Se)$.

Compound	Shielding	$\delta(^{77}Se)$ Meas./ppm
B	1643.2	62
A	1549.3	119
C	1686.9	35
D	1558.5	135
F	1320.0	304
G	1330.1	322
H	1145.6	474
I	1404.1	158
I	1615.1	97
I	2015.7	-286
J	1559.3	105
K	1580.1	100
L	1407.3	254
M	1697.5	-31
N	1845.2	-146
B	2238.6	-516
A	1762.3	-79
D	1576.2	122
E	1831.0	-163
O	1745.0	-23
$SeMe_2$	1607.8	0

Table S2. mPW1PW91/6-31+G(d,p) calculated isotropic chemical shieldings *v.* measured δ(^{77}Se).

Compound	Shielding	δ(^{77}Se) Meas./ppm
B	1701	62
A	1610.6	119
C	1741.1	35
D	1608.4	135
F	1443.1	304
G	1417.9	322
H	1209.1	474
I	1488.1	158
I	1673.8	97
I	2062.4	-286
J	1659.2	105
K	1647.3	100
L	1474.9	254
M	1774.8	-31
N	1910.4	-146
B	2312.7	-516
A	1809.7	-79
D	1643.4	122
E	1888.2	-163
$SeMe_2$	1725.0	0

Table S3. Calculated MP2/6-31+G(d,p) isotropic chemical shieldings *v.* measured δ(^{77}Se).

Compound	Shielding	δ(^{77}Se) Meas./ppm
B	1791.8	62
A	1735	119
C	1862.8	35
D	1683.9	135
F	1557.4	304
G	1529.6	322
H	1387.4	474
I	1643.9	158
I	1828.8	97
I	2201.3	-286
J	1792.6	105
K	1791.1	100
L	1642.6	254
M	1883.4	-31
N	2037.1	-146
A	2438	-516
B	1968.2	-79
D	1754.9	122
E	2024.6	-163
$SeMe_2$	1888.8	0

Table S4. MP2 calculated Cartesian coordinates for $Se_2B_{18}H_{20}$ **A**.

B	-2.8201887935	0.9266123815	-1.1094917516
B	-0.5764515809	0.1807232505	1.4652028032
B	1.8743320792	1.7848403874	0.9536407921
B	-1.0257184799	1.8594825433	0.9080111393
B	3.6521254738	-0.0789918534	-0.5452683455
B	-2.1974259264	-0.7942140692	1.2873432262
B	2.2139696765	-0.7719679591	-1.4973901915
B	0.6507678087	-0.7308722508	-0.4264940887
B	3.2891724205	1.5864424304	-0.1088826506
B	-2.2387498917	0.9272831893	1.7507218389
B	-1.3258486156	1.952638911	-0.7949965588
B	2.7336971684	0.9045208671	-1.6561990062
B	0.9805713867	0.4986016695	-1.5937211507
H	-3.5359535335	1.196159175	-2.0141185172
H	-1.1092327393	2.9061609643	-1.4600614743
H	4.1730614396	2.37706537	-0.0874118741
H	-2.5323753743	1.2260458618	2.8601124175
H	-2.4786257168	-1.6524194148	2.0550968828
H	2.3665896315	-1.6540679537	-2.2687560537
H	4.7341265951	-0.5416254407	-0.6533869782
H	0.0501773491	-0.0109251312	2.4547607776
H	3.2229316046	1.2285347644	-2.6862868862
H	0.3628173482	0.5644913411	-2.6026794655
H	1.3934636842	3.0546830027	-1.134818886
B	-3.5022572987	0.4071102037	0.6336689153
Se	-2.8916009037	-1.1076453179	-0.6461059829
H	-0.5601290068	2.7900309385	1.4689543735
H	-0.0992154487	-1.6204409646	-0.6223490457
H	-4.6373888799	0.3552736643	0.9620629962
H	-0.9114373549	-0.9963991392	1.0396231764
H	-1.564224158	0.9790624537	-1.6005126078
B	-2.6932709296	1.9403494601	0.354653448
H	-3.3226392125	2.9324818601	0.5141987834
Se	2.3570550645	-1.4316052107	0.5234908557
B	3.0783980218	0.4513336201	1.2226462711
B	0.2872946901	1.037374116	-0.0064395663
B	1.6475071347	1.964306109	-0.7504206157
H	1.7750759724	2.7035547633	1.6922847609
H	3.8132654396	0.3246733794	2.1417735339
H	1.8922898559	0.7603830276	1.7448007053

Table S5. MP2 calculated Cartesian coordinates for $[Se_2B_{18}H_{19}]^-$ anion **B**.

B	-2.7565873826	1.0949304071	1.0162111094
B	-0.6208266822	-1.6840477833	-0.2094311035
B	1.7512491317	-0.0792152657	-1.6588267212
B	0.6132275073	-0.4784266195	-0.2121440317
B	-0.3843429765	-0.586581893	1.3705490735
B	3.4112076915	0.4082558137	-0.9043950068
B	-2.37903595	-1.4873763074	-0.610738745
B	2.2289254623	1.594191563	-1.4045259545
B	0.5740418831	1.0712784992	-1.0458884998
B	3.3296929471	0.2999499487	0.9933924554
B	-1.8627709873	-1.6754993866	1.058471167
B	0.4354223818	0.9425473653	0.7199778249
B	-1.0450642662	1.176032753	1.6047960623
B	3.1300962986	1.8131313561	0.1305826363
B	1.3729510137	2.2337602584	0.0276678345
B	2.0295941307	1.4660556476	1.4779487286
H	-3.5633981431	1.8609668188	1.433303462
H	-0.9116167772	1.8805221627	2.5581718006
H	4.3001434622	0.0111993882	1.6103647251
H	-2.0051576868	-2.7041649946	1.6392707477
H	-2.9492446271	-2.3489546335	-1.1976833315
H	2.485513037	2.2577068604	-2.3563045046
H	4.4289494742	0.1527993843	-1.4544708175
H	-0.2314207185	-2.8020517327	-0.3718800229
H	3.9869731238	2.6332518737	0.2038994731
H	1.0527859661	3.377607472	0.1217434608
H	2.1152659432	2.0826745532	2.491727264
H	2.1950772281	0.2371679853	1.7663398371
B	-3.2954839936	-0.7047174656	0.843099099
Se	-3.2378176746	0.4087696487	-0.9211669584
H	1.7257220056	-0.6618856884	-2.6912706338
H	0.295290706	-1.059918832	2.2397095853
H	-0.3180775735	1.3629897952	-1.7773988791
H	-4.3703995702	-1.0817224827	1.1767031936
H	-1.2048850769	-1.256788533	-1.2697733194
H	-1.6408176656	1.8025633789	0.658553971
Se	2.5162585486	-1.3358497393	-0.0779672037
B	-2.0899417152	-0.20197816	1.9931897278
H	-2.3749344756	-0.3364004164	3.1405754946

Table S6. MP2 calculated Cartesian coordinates for $[Se_2B_{17}H_{18}]^-$ anion **C**

B	-0.0015850126	2.6102399722	-0.0842065255
B	0.0015850126	-2.6102399722	-0.0842065255
B	0.3752916539	1.6585990371	1.3523516084
B	-0.3752916539	-1.6585990371	1.3523516084
B	-0.9972262824	2.7810172281	1.4096957552
B	0.9972262824	-2.7810172281	1.4096957552
B	-1.6893963075	3.0603128161	-0.1908425913
B	1.6893963075	-3.0603128161	-0.1908425913
B	0.4296260674	0.8494720682	-0.3006721425
B	-0.4296260674	-0.8494720682	-0.3006721425
B	0.	0.	1.1898853269
B	-1.1332292551	1.1662907729	2.1007611679
B	1.1332292551	-1.1662907729	2.1007611679
B	-2.4998969024	1.8925455163	1.1187911521
B	2.4998969024	-1.8925455163	1.1187911521
B	-0.8747416566	1.7758781228	-1.311869078
B	0.8747416566	-1.7758781228	-1.311869078
H	0.8306961169	3.3883021087	-0.4311452359
H	-0.8306961169	-3.3883021087	-0.4311452359
H	1.3135308753	1.9233681326	2.0367859953
H	-1.3135308753	-1.9233681326	2.0367859953
H	-0.9279244284	3.7351020504	2.1146763033
H	0.9279244284	-3.7351020504	2.1146763033
H	-2.0810331452	4.1387562411	-0.4903002419
H	2.0810331452	-4.1387562411	-0.4903002419
H	1.5097060548	0.8524255526	-0.7894734132
H	-1.5097060548	-0.8524255526	-0.7894734132
H	-1.3136984868	1.0284235229	3.2672566023
H	1.3136984868	-1.0284235229	3.2672566023
H	-3.5147923614	2.1408517443	1.6817441102
H	3.5147923614	-2.1408517443	1.6817441102
H	-0.6771877363	1.9426846645	-2.4723585673
H	0.6771877363	-1.9426846645	-2.4723585673
H	-2.0353307063	0.5843477387	1.4237774124
H	2.0353307063	-0.5843477387	1.4237774124
Se	-2.8189065861	1.4555831533	-0.8751215219
Se	2.8189065861	-1.4555831533	-0.8751215219

Table S7. MP2 calculated Cartesian coordinates for $Se_2B_{17}H_{17}$ **D**

Se	2.0001837853	-1.4125751839	0.2727164354
Se	-2.9108020613	-0.0902386212	-0.9660604544
B	1.86387155	1.040114017	-1.8290843294
B	-1.9081286289	0.201994954	2.0738911883
B	1.3321856624	2.2016963381	-0.5880983251
B	-0.1855526421	0.346781486	1.8242358056
B	0.238279761	0.9057272088	-1.1390466051
B	-1.002546209	-1.1565156116	1.2843110398
B	1.1961071868	-0.551748215	-1.5164196388
B	-2.7072624298	-0.8877112337	0.9384644144
B	2.9681216317	-0.1110096903	-1.1148077191
B	-1.2204735927	1.7298625608	1.453662742
B	3.0137710339	1.5593559888	-0.5548830529
B	0.3173758838	1.3743414001	0.5457124762
B	0.1652288748	-0.3566655728	0.148203711
B	-1.4320722486	-1.2959370765	-0.3714698143
B	3.1282376965	0.3527155263	0.7173774644
B	-2.7901736586	1.0261650626	0.7719916223
B	2.1025876091	1.8368928174	0.9394509403
H	2.0068279699	1.3366455749	-2.9677431537
H	-2.2905660724	0.1144161505	3.1921222636
H	1.1297503223	3.3465662429	-0.8276677124
H	0.5072667807	0.2828519397	2.7872195108
H	-0.6781117096	1.1338138169	-1.8499547815
H	-0.6283589528	-2.1377848194	1.8388196102
H	0.9200096464	-1.3837327382	-2.3074155522
H	-3.5772074978	-1.5917710985	1.3198757237
H	3.8506835773	-0.6807293555	-1.6558095888
H	-1.3307586504	2.7032294547	2.1208565043
H	3.956498893	2.2116609806	-0.8543481829
H	-1.3089379577	-2.302901157	-0.9819494498
H	4.108177079	0.0820210274	1.3211898652
H	-3.7872128572	1.6335271705	0.9675551711
H	2.39640721	2.6824716674	1.7162032306
H	-1.7521084717	1.8631093268	0.3000851452
H	2.156911487	0.7234556618	1.5723614958

Table S8. MP2 calculated Cartesian coordinates for $[SeB_{18}H_{21}]^-$ anion **E**

B	-2.3881576104	-1.4415137105	-0.7106706195
B	1.5464080347	1.1088317296	1.6316613495
B	-1.4936100245	-0.1299966636	-1.4848104303
B	0.3662188539	1.9245633883	0.6255312302
B	-3.2506571794	-0.0293977702	-1.3296830958
B	2.0707571032	1.9872793489	0.1522144381
B	-3.9057919344	-0.9225979625	0.064130161
B	2.9376169515	0.5557918051	0.7381912533
B	-0.8407177204	-0.9182354701	0.0347727435
B	-0.0434293252	0.2773490826	1.2986623148
B	-0.6717396527	0.8677405567	-0.2916048361
B	-2.2895980362	1.3791520666	-0.9822675898
B	0.8136377759	1.7524489074	-1.0600597344
B	-3.8478360503	0.8758667028	0.0530206891
B	2.3600281958	0.7786506534	-1.1041035415
B	-2.4682296592	-1.3964750916	1.0244515279
B	1.5952679531	-0.5964288908	1.383990279
H	-2.3559330553	-2.5180753669	-1.2158712361
H	1.6861787124	1.5300057668	2.7363515668
H	-1.0228147392	-0.2890151233	-2.5650397908
H	-0.1576851243	2.9372278416	0.9712797958
H	-3.8984806458	0.0302138113	-2.3259710095
H	2.6498872666	3.0229844502	0.2074713349
H	-4.8974541069	-1.5632880032	-0.0584163266
H	4.0578292505	0.6578584942	1.1148193139
H	-0.1007609399	-1.8041700309	-0.220075215
H	-0.6567640416	0.3486912655	2.317012971
H	-2.32661781	2.3614824527	-1.6487729566
H	0.6332667394	2.5740086984	-1.8992120244
H	-4.8004807452	1.5831647427	0.0104827646
H	3.1107048924	0.9606830557	-2.0056365907
H	-2.3928834176	-2.35236568	1.725868238
H	1.7972585635	-1.3725275508	2.2624805011
H	-1.7507226544	1.6447416727	0.1282070567
H	1.0529836778	0.6283971403	-1.5982710787
B	-3.5728902533	-0.1017406401	1.5320926484
Se	2.4871629802	-1.1464052744	-0.3609153226
H	-2.3221744997	-0.351569406	1.8102411276
H	-4.2121330686	-0.0726640391	2.5285668312
H	-3.2642446567	1.1391330408	1.2103812617

Table S9. MP2 calculated Cartesian coordinates for $[SeB_{18}H_{19}]^-$ anion **F**

B	-2.1505172072	-0.8427197561	-1.3444322595
H	-2.6099002738	-1.4234540416	-2.2728597851
B	-1.136350547	-1.6636196789	-0.1348953904
B	-0.4222225061	-0.496397467	-1.2777825528
B	-1.5477969482	0.8079274165	-1.574943053
B	-3.1065415065	0.4104079193	-0.5902610695
B	-2.783670284	-1.0862704791	0.3033716506
H	-0.9272448935	-2.8330883127	-0.1876544497
H	0.2157739567	-0.8419009905	-2.2194657981
H	-1.6429201783	1.422495597	-2.5839629087
H	-4.1787375834	0.7455431429	-0.9652429478
H	-3.6910738708	-1.8308572531	0.4824127178
B	0.1369246042	-0.576190955	0.4474140499
B	-0.0127050924	0.9844089067	-0.4787901104
B	-2.6287396406	0.3788829364	1.2580682622
B	-1.4275208278	-0.9715217912	1.448292511
H	-3.4057149146	0.7137427262	2.08883551
H	-1.4050098001	0.2469871957	1.8309356248
H	-1.4111196884	-1.6392095642	2.4309957413
B	1.6538371383	-0.4796557088	1.4067557836
B	0.7284561597	1.0069977393	1.1349406732
B	1.4244890185	1.8436826665	-0.2807497685
B	1.8825074831	-1.6503408938	0.1509767831
H	1.6281034651	-0.9332312687	2.5079778195
H	0.1852444857	1.5204887631	2.0652587333
B	2.4318283854	1.0719090071	0.9269509952
B	3.1709832577	-0.4890664718	0.4483526434
H	1.4940235221	3.0367566699	-0.3410676686
B	2.8627611434	0.8241764195	-0.7937487668
H	1.8783818295	-2.8180435186	0.371807966
H	3.1173979417	1.7241554302	1.6538454503
H	4.2128356552	-0.7676294339	0.948902793
H	3.775227964	1.3810142018	-1.3215577272
Se	-1.956041723	2.0178833634	0.1119291775
H	3.5383341698	-1.5067368317	-1.9234136907
H	2.2470248828	0.1143559694	-1.7190005632
H	1.6741605634	-1.5407931569	-1.1487435129
B	2.8292051192	-0.9390475177	-1.1610285227

Table S10. MP2 calculated Cartesian coordinates for $SeB_{18}H_{20}$ **G**

B	-1.547826311	1.6208929233	1.291400328
H	-1.8877880526	2.3139836045	2.1896103205
B	-0.3791604892	2.1677398787	0.0688779133
B	0.0758210198	0.9270623995	1.2716669824
B	-1.3155927531	-0.0976154329	1.6046510552
B	-2.7196120667	0.5475439755	0.5578322955
B	-2.0960959417	1.9136209606	-0.3814618943
H	0.0819898104	3.2596787854	0.0836404432
H	0.7671176325	1.1322402272	2.2113471827
H	-1.5002563705	-0.6642428872	2.6257275171
H	-3.839898933	0.4178616549	0.9098580099
H	-2.8357778252	2.811644334	-0.6025751273
B	0.6815693457	0.8439542048	-0.4437962024
B	0.1246855839	-0.634474252	0.4814350458
B	-2.2180497074	0.4105066446	-1.2829778721
B	-0.7731927422	1.4938615928	-1.4976234424
H	-3.0300042853	0.1824911953	-2.1124156962
H	-0.993203715	0.279945603	-1.8427276043
H	-0.6010287639	2.1026486856	-2.4993753501
B	2.1269407121	0.4099236625	-1.3923866936
B	0.9272434211	-0.8560463143	-1.0660529004
B	1.479591301	-1.8187779378	0.3306591399
B	2.5654753053	1.5254163316	-0.1298658531
H	2.1710052299	0.8280641228	-2.501791722
H	0.3248484484	-1.3224083881	-1.9753533628
B	2.6186388817	-1.2158972949	-0.8702636968
B	3.6493609849	0.160140031	-0.4711500455
H	1.274178662	-2.9828907874	0.4178905827
B	3.2153128761	-1.0813380618	0.7772169581
H	2.7570775869	2.674358155	-0.3417725145
H	3.0646269743	-2.0762931547	-1.5549103287
H	4.6974306268	0.2432520905	-1.0146665175
H	3.9942857665	-1.8308315051	1.2610810584
Se	-1.9027182823	-1.2981828513	-0.0582618974
H	4.2268442475	1.1059995348	1.9125391529
H	2.7646922189	-0.3147534986	1.7488605674
H	2.3874530974	1.4363436356	1.1691284635
B	3.4373204094	0.6637631885	1.1526186376
H	0.9724980966	-1.262822056	1.3747200677

Table S11. MP2 calculated Cartesian coordinates for $SeB_{17}H_{19}$ **H**

B	0.9026323606	0.7469419762	1.7329427029
B	-0.2625051466	1.600821845	0.6583627856
B	1.4599901515	1.9731407457	0.5188268198
B	2.5242059761	0.654645067	1.0115771847
B	-0.3604295593	-0.1668612717	0.9000427634
B	0.4808687123	1.7848449412	-0.9463266397
B	2.27203055	1.155240014	-0.7681124138
B	1.4037819579	-0.8162723468	1.1972462857
H	0.7762051526	0.9264119501	2.8990916146
H	-0.9651376042	2.4070122934	1.1669125596
H	1.7759359571	3.0877901439	0.7741497222
H	-1.3149565381	-0.3010961057	1.8242806736
H	0.2726207043	2.699774855	-1.6678322814
H	3.0936057332	1.6309125301	-1.4747895349
H	1.5264017766	-1.7640657626	1.8951080284
B	-2.0342770626	-0.3687034235	-1.4824166687
B	-0.8762098397	-1.2112244185	-0.413256523
B	-2.5962963563	-1.5648784149	-0.2849679026
B	-3.6773795281	-0.2404433206	-0.7748468132
B	-0.8039202459	0.5724255711	-0.6623247406
B	-1.659569304	-1.3891651604	1.1888242637
B	-3.4905077262	-0.7049492271	0.9481011839
B	-3.7056827131	0.9903007963	0.4282338023
B	-2.6473561717	1.1900928301	-0.9773524812
H	-1.9153133994	-0.5210313408	-2.6529845083
H	-0.1668207502	-2.0243974329	-0.8924669255
H	-2.9382656884	-2.6799010185	-0.5013364549
H	0.2276892441	0.6760103848	-1.5548805911
H	-1.4750538276	-2.297920329	1.9241612357
H	-4.3326878585	-1.1982475549	1.6171207604
H	-3.1722789391	0.4304703135	1.5162509492
H	-4.5688910735	1.7191922465	0.7728490105
H	-2.6174896584	1.7068713195	0.2238914536
H	-2.7907473823	2.0944146421	-1.7272471431
H	3.524202424	0.8170653052	1.6184926436
H	-4.6229811592	-0.493546317	-1.438869306
Se	2.4867988323	-0.8225033258	-0.4752685147

Table S12. MP2 calculated Cartesian coordinates for $[Se_3B_{18}H_{21}]^-$ anio**n I**

H	-5.6294541308	0.6625056428	1.4575882202
H	-2.8913228001	-0.8438045699	2.4228086499
H	-3.1488316835	2.1266129968	2.3882590276
H	-5.445245948	0.4995106149	-1.6940588193
H	-4.7076364543	2.9730935436	-0.2074679809
H	4.0411172011	-2.7898576539	1.2409713275
H	-0.6827468766	1.244049677	1.6895856691
H	1.4516792338	-2.6797300359	-0.2930196027
H	2.574261232	0.2227002367	1.6635310909
H	-1.8775377143	3.364233625	0.0734727977
H	-2.3610690579	0.5437420988	-1.7513150291
H	4.1051072233	-2.5058142618	-1.7035312839
H	-3.0795661431	2.2807743136	-2.4846334674
H	5.9894274226	-1.0053654694	-0.0291985093
H	-0.442886493	1.575845542	-1.5965824556
H	1.7989113018	-0.954286803	-2.6365384696
H	2.6488247194	0.5397650966	-1.6067523967
H	4.5415902041	0.3851257804	-2.4658875044
B	-4.6103290264	0.6550146041	0.8552685303
B	-3.040696411	-0.1918305463	1.4440812361
B	-3.1403801328	1.5164060314	1.3685794917
B	-4.4835139984	0.5917740752	-1.0047882045
B	-4.027921079	2.0015250746	-0.1138516616
B	-1.5247168807	0.9253586321	0.9150285256
B	2.2039619135	-0.9620112232	1.3893758023
B	3.6478046163	-1.7842998826	0.745495634
B	-2.2808615186	2.2519076154	-0.0288267228
B	2.1351978508	-1.7083703602	-0.1839117312
B	3.8658406188	-0.2189104739	1.5214864255
B	-3.0252380232	1.6089719012	-1.506557484
B	1.2376666189	-0.2459541988	-0.0637090231
B	-1.3474399712	1.1140414833	-0.9852907497
B	3.687703962	-1.6113654408	-1.0424842295
B	4.8311928283	-0.749622115	-0.0302301272
B	2.2724554491	-0.6738969289	-1.581986746
B	3.9353960334	0.0727063197	-1.4942952257
Se	-4.0246544057	-1.1393238622	-0.0407798588
Se	-0.7511847779	-0.7066910722	-0.1615236082
Se	4.4035579624	1.2852075692	0.1558806574
H	1.6842015316	-1.4361395257	2.3483852079
H	4.4261400677	-0.0999253896	2.5610722707
H	1.4045835353	0.927291339	0.0556103269

Table S13. MP2 calculated Cartesian coordinates for $[SeB_{10}H_{11}]^{-}$ anion **J**

B	-0.1784030393	0.8246596612	1.2962041932
B	-0.1812616424	1.5965862412	-0.4462084329
B	-0.1540188937	-1.0636447316	1.1330686082
B	1.2831038677	-0.0925879909	1.4518566476
B	1.2789426972	1.4196293778	0.5077959383
H	-0.7688104805	1.3678602921	2.1702418275
B	1.3520615421	0.9964932335	-1.2338267241
B	1.3559794704	-0.8239782269	-1.3470831506
B	-0.1047277946	-1.476550494	-0.710177451
B	1.3368819255	-1.470500506	0.315496476
B	2.2260614157	-0.0092548777	-0.0649721838
H	-0.7419930654	-1.6978991313	1.9470030956
H	1.7869694947	2.4045494862	0.9436458494
H	1.7739036874	-0.1273922839	2.5358673534
H	0.1417769975	0.9795475586	-1.6246599825
H	-0.7218835141	2.6408065939	-0.6130763297
H	3.4156597422	0.0910774392	-0.0413638185
H	1.8507925291	-2.5020289852	0.625775511
H	-0.6877222805	-2.4714786134	-1.0170134993
H	1.9994396611	-1.3783123346	-2.1915595128
H	1.949468429	1.7341258214	-1.9566322765
Se	-1.5051837489	-0.0118555298	-0.1627931386

Table S14. MP2 calculated Cartesian coordinates for $SeB_{10}H_{12}$ **K**

B	-1.0504592906	0.6716507606	0.9319221902
B	0.575394322	-0.104048763	1.5582964819
B	-1.0504592906	0.6716507606	-0.9319221902
B	-1.863334881	-0.5598427212	0.
B	-0.9299809426	-1.0059026986	1.4602585609
H	-1.5380506661	1.5535287437	1.5489670743
B	0.5739572863	-1.8287745216	0.9497032389
B	0.5739572863	-1.8287745216	-0.9497032389
B	0.575394322	-0.104048763	-1.5582964819
B	-0.9299809426	-1.0059026986	-1.4602585609
B	-0.8815794919	-2.0247048967	0.
H	-1.5380506661	1.5535287437	-1.5489670743
H	-1.4911256609	-1.3190996859	2.4555589791
H	-3.0481243674	-0.57441843	0.
H	1.4707843936	-0.9058285897	0.9463695979
H	1.0684017324	0.2841742756	2.5604104669
H	-1.3437463042	-3.1173508554	0.
H	-1.4911256609	-1.3190996859	-2.4555589791
H	1.0684017324	0.2841742756	-2.5604104669
H	1.0234312755	-2.70893915	-1.602830132
H	1.0234312755	-2.70893915	1.602830132
Se	0.7473051475	1.3381861607	0.
H	1.4707843936	-0.9058285897	-0.9463695979

Table S15. MP2 calculated Cartesian coordinates for $SeB_{11}H_{11}$ **L**

Se	0.0000000303	0.0001272586	-1.5071313326
B	0.0000000175	1.6219415685	-0.1229459098
B	1.5424880312	0.5011949328	-0.123071063
B	0.9532992565	-1.3121407976	-0.1230528891
B	0.8936994175	1.2300564108	1.3399246689
B	-0.8936993296	1.2300564671	1.3399247071
B	-1.446013588	-0.4698390479	1.3398932211
B	1.4460135363	-0.4698391048	1.3398931074
B	-0.0000000231	-1.5204173676	1.3399111514
B	-0.000000083	0.0000179339	2.2649979362
H	-0.0000000923	0.0000287751	3.4510650267
H	-0.0000000334	-2.5934485753	1.844157004
H	-2.4665333416	-0.8014409527	1.8441217442
H	2.4665332655	-0.80144102	1.8441216729
H	1.5244222041	2.0981608636	1.8441417752
H	1.5528774422	-2.1373232844	-0.7196538277
H	2.5125191738	0.8162281611	-0.7199161812
H	0.0000000136	2.6419575659	-0.71952204
B	-0.9532992544	-1.3121407102	-0.1230527652
H	-1.5528774721	-2.1373231794	-0.7196536928
H	-1.5244221005	2.0981609541	1.8441417788
B	-1.5424879418	0.5011949136	-0.1230710307
H	-2.5125190508	0.8162281221	-0.719916206

Table S16. MP2 calculated Cartesian coordinates for $SeB_{11}Cl_{11}$ **M**

Se	0.0004651564	-0.0000800004	-2.1291397221
B	-0.9654201459	1.3293762534	-0.7096789547
B	0.9667331374	1.3298358012	-0.7087604331
B	1.5627627617	-0.5074360158	-0.7092737897
B	0.000232806	1.5311367074	0.7585803264
B	-1.4569558609	0.4735029823	0.7587969259
B	-0.9007259831	-1.2393568661	0.7585341126
B	1.4562178767	0.4731456815	0.7585580635
B	0.8995208149	-1.2390011249	0.7585141191
B	-0.0002249498	-0.0002423514	1.6819403027
B	-0.0001948496	-1.6424221625	-0.7093108488
B	-1.5625099993	-0.5077498011	-0.7091843578
Cl	-2.9479267295	0.9585529419	1.5620992502
Cl	-0.0002309994	3.0994879367	1.5616883257
Cl	-1.8900923344	2.5996431509	-1.4703505483
Cl	1.8905803215	2.6019379153	-1.4673777025
Cl	3.0567565951	-0.9940009189	-1.4693493535
Cl	0.0012800938	-3.2139962756	-1.4687875192
Cl	1.8217826901	-2.5075396967	1.5613987496
Cl	-1.8220197943	-2.5084813992	1.5614290707
Cl	-0.0000137383	-0.0003511818	3.4394384038
Cl	-3.0576023766	-0.9939776433	-1.4679844305
Cl	2.9475855076	0.9580160671	1.5621927099

Table S17. MP2 calculated Cartesian coordinates for SeB_5Cl_5 **N**

Se	-0.7748017569	0.90126525	0.021510275
B	1.0066819038	0.753581176	0.043020996
B	0.0326292627	-0.0618757777	1.1693357004
B	0.8578493171	-1.0280714712	0.037124279
B	-0.9241331512	-0.8802389611	0.0304964059
B	0.0199559613	-0.0581659837	2.9129813089
Cl	-2.2545329264	-2.0067374198	0.0199084533
Cl	2.3378267575	1.8793063694	0.0507585223
Cl	-1.9003139736	2.2329045521	0.0179581154
Cl	1.9840743499	-2.3590424812	0.0555601393
Cl	0.0547659358	-0.0662107728	-1.6574744553

Table S18. DFT/B3LYP calculated Cartesian coordinates for $Se_2B_{18}H_{20}$ Compound **A**

B	-2.857827681	0.9045268464	-1.0933681122
B	-0.5865102956	0.1671328035	1.4414961144
B	1.8743933727	1.7730006816	0.9635661365
B	-1.0184502388	1.8555108644	0.8891173531
B	3.6695531925	-0.0606533303	-0.5363808812
B	-2.2281043008	-0.7838322854	1.2938672263
B	2.2467795463	-0.7530968394	-1.5060461685
B	0.678247912	-0.7396215406	-0.4398383705
B	3.2937969198	1.5986867208	-0.0921610732
B	-2.228666155	0.9442393133	1.7511338261
B	-1.3349637613	1.9081812977	-0.8152594482
B	2.7543971061	0.9274835344	-1.6481579439
B	1.004198396	0.5059180873	-1.5973708828
H	-3.5623175896	1.1972850973	-1.9997399904
H	-1.1221383163	2.8309748748	-1.5242473166
H	4.173491831	2.3949494221	-0.0546520895
H	-2.5075427189	1.2546733111	2.8622694345
H	-2.4930925513	-1.6200073674	2.0905284995
H	2.4112719156	-1.6188309359	-2.2936920569
H	4.7579295875	-0.5074487517	-0.6482730179
H	0.0358272792	-0.0579478466	2.4255267526
H	3.2470204024	1.2607396423	-2.674716933
H	0.3890758063	0.5757122454	-2.6082248151
H	1.3932185443	3.0637730098	-1.118063941
B	-3.5187079953	0.4372922501	0.6650350647
Se	-3.0313834261	-1.1338560226	-0.6104481187
H	-0.5510908561	2.7930083359	1.4375851808
H	-0.0759374913	-1.623880813	-0.6500279485
H	-4.6454378312	0.4283228058	1.0281989031
H	-0.9604069433	-0.9884421808	0.9756192867
H	-1.5892188543	0.8878669553	-1.5610985149
B	-2.6872986951	1.9482126052	0.346373386
H	-3.3005407151	2.9534568398	0.4967338881
Se	2.393688717	-1.4661278319	0.5116432828
B	3.0881772565	0.4447423395	1.2248005379
B	0.2944263345	1.0302093842	-0.0161221479
B	1.6551296381	1.9716367978	-0.7403587857
H	1.763111401	2.6762012103	1.7198939929
H	3.8161121106	0.3243249843	2.1495393017
H	1.8857451472	0.724728485	1.7225763895

Table S19. DFT/B3LYP calculated Cartesian coordinates for $[Se_2B_{18}H_{19}]^-$ anion B.

B	-2.8006445739	1.0936061397	1.0077522968
B	-0.6326122073	-1.6855755504	-0.223909704
B	1.7773132431	-0.068156441	-1.6665436414
B	0.6060583205	-0.485999932	-0.2178178056
B	-0.3998063544	-0.5832554344	1.3805220692
B	3.4463407227	0.4234386302	-0.9154836608
B	-2.420871879	-1.5054193113	-0.6094437361
B	2.2560295444	1.6026040276	-1.4030104471
B	0.5998576671	1.075219899	-1.0514747284
B	3.3712074737	0.2957551835	0.9901456468
B	-1.8708195383	-1.678934622	1.0591797075
B	0.4351302718	0.9381725316	0.718108619
B	-1.0610866435	1.1882834895	1.5851504833
B	3.1624639913	1.8148148337	0.1390938601
B	1.3947183449	2.2241793592	0.0408933581
B	2.0592280452	1.4632271121	1.4863771725
H	-3.5987241797	1.8646786268	1.4340599632
H	-0.9426552527	1.9217125913	2.5211000573
H	4.3417078858	0.0062285717	1.607876634
H	-2.0060309764	-2.706055494	1.6509147098
H	-2.981701511	-2.3842032459	-1.181596027
H	2.5079307181	2.27970795	-2.3491208672
H	4.4647227356	0.1730706892	-1.4677916109
H	-0.2556458419	-2.8074982559	-0.4044033559
H	4.0142722691	2.6443401038	0.212205726
H	1.0745513219	3.3678146822	0.142897505
H	2.1435340179	2.0788935403	2.503281355
H	2.2653828866	0.2425591626	1.7955423238
B	-3.3154341283	-0.7222750592	0.8661113923
Se	-3.3482290907	0.3824293415	-0.9296951055
H	1.7511357619	-0.6447638234	-2.7020518526
H	0.2704211756	-1.0518338507	2.2537920336
H	-0.2920396492	1.376976197	-1.7812887831
H	-4.38141002	-1.108350327	1.2194932458
H	-1.2663447722	-1.2687068607	-1.2764464548
H	-1.6996614434	1.7927899012	0.634844692
Se	2.5599389196	-1.3530287719	-0.0981216463
B	-2.1052185594	-0.190402999	1.9947177507
H	-2.3864446955	-0.3232695851	3.1465218249

Table S20. DFT/B3LYP calculated Cartesian coordinates for $[Se_2B_{17}H_{18}]^-$ anion **C**

B	-0.0189112167	2.6250270979	-0.0901357395
B	0.0189112167	-2.6250270979	-0.0901357395
B	0.3443810866	1.6567567839	1.3502659529
B	-0.3443810866	-1.6567567839	1.3502659529
B	-1.0239521265	2.7954269707	1.4161131638
B	1.0239521265	-2.7954269707	1.4161131638
B	-1.6987699773	3.1007979171	-0.18849894
B	1.6987699773	-3.1007979171	-0.18849894
B	0.4201885418	0.8599922491	-0.3117744507
B	-0.4201885418	-0.8599922491	-0.3117744507
B	0.	0.	1.1789861003
B	-1.1727760343	1.180131359	2.1001777206
B	1.1727760343	-1.180131359	2.1001777206
B	-2.5396856118	1.9313099038	1.1184527808
B	2.5396856118	-1.9313099038	1.1184527808
B	-0.8953362956	1.8205957051	-1.3220553229
B	0.8953362956	-1.8205957051	-1.3220553229
H	0.8263585235	3.3996690253	-0.4187667971
H	-0.8263585235	-3.3996690253	-0.4187667971
H	1.2732783464	1.9224377901	2.0483783767
H	-1.2732783464	-1.9224377901	2.0483783767
H	-0.9346911121	3.7482403332	2.1251652937
H	0.9346911121	-3.7482403332	2.1251652937
H	-2.0821399828	4.184389878	-0.4847682741
H	2.0821399828	-4.184389878	-0.4847682741
H	1.5019012131	0.8634426546	-0.7976801996
H	-1.5019012131	-0.8634426546	-0.7976801996
H	-1.3443228173	1.0400307229	3.2696579169
H	1.3443228173	-1.0400307229	3.2696579169
H	-3.5519265889	2.1916989343	1.6819548702
H	3.5519265889	-2.1916989343	1.6819548702
H	-0.696746013	1.9827354481	-2.4832688682
H	0.696746013	-1.9827354481	-2.4832688682
H	-2.1072507583	0.6178511824	1.448840969
H	2.1072507583	-0.6178511824	1.448840969
Se	-2.8640260146	1.5087311665	-0.8967582955
Se	2.8640260146	-1.5087311665	-0.8967582955

Table S21. DFT/B3LYP calculated Cartesian coordinates for $Se_2B_{17}H_{17}$ **D**

Se	2.0484293572	-1.4405029783	0.2655881796
Se	-2.9820512798	-0.1131943413	-0.9704293069
B	1.8897156319	1.0449804462	-1.8435220239
B	-1.9421790399	0.2152902889	2.0790232389
B	1.345321446	2.1990420572	-0.5821017333
B	-0.2092931174	0.3422331959	1.8241512857
B	0.2500759947	0.8917180244	-1.148641162
B	-1.0324027638	-1.1755716027	1.2760887193
B	1.2319201228	-0.5576583722	-1.5468045906
B	-2.7523733342	-0.8996001713	0.9697194981
B	3.016658117	-0.1027811088	-1.1461605807
B	-1.2400141513	1.7445917321	1.4436704744
B	3.049047906	1.5659105663	-0.5531729496
B	0.3225069435	1.3617489814	0.549004189
B	0.1707780772	-0.3826495272	0.1458020052
B	-1.4849788222	-1.3274288163	-0.3696920842
B	3.1822110825	0.3423860921	0.7162282723
B	-2.8477407697	1.0325912079	0.7743895487
B	2.1276445639	1.8428387082	0.9476993491
H	2.0321787978	1.3647384407	-2.9746467429
H	-2.3108608433	0.1285687728	3.2013560545
H	1.1391743536	3.3410629713	-0.819290293
H	0.4688079377	0.2875641617	2.7933114297
H	-0.6695298417	1.116593612	-1.8526283723
H	-0.6607664312	-2.1473252962	1.8451880017
H	0.9572851713	-1.3862347855	-2.3379189613
H	-3.618864607	-1.6019512449	1.3555595312
H	3.9033633807	-0.6628310184	-1.6861434946
H	-1.333046475	2.7092434044	2.1250614944
H	3.9800774516	2.2335340892	-0.8537064393
H	-1.3639018561	-2.3313450799	-0.9819032863
H	4.1751557588	0.0868255159	1.3023934593
H	-3.8424407656	1.6344448943	0.9868590669
H	2.4197501243	2.704222282	1.7061985271
H	-1.8308085053	1.9214848301	0.3181458719
H	2.2593603853	0.747556068	1.6228718235

Table S22. DFT/B3LYP calculated Cartesian coordinates for $[SeB_{18}H_{21}]^{-}$ anion **E**

Se	2.0484293572	-1.4405029783	0.2655881796
Se	-2.9820512798	-0.1131943413	-0.9704293069
B	1.8897156319	1.0449804462	-1.8435220239
B	-1.9421790399	0.2152902889	2.0790232389
B	1.345321446	2.1990420572	-0.5821017333
B	-0.2092931174	0.3422331959	1.8241512857
B	0.2500759947	0.8917180244	-1.148641162
B	-1.0324027638	-1.1755716027	1.2760887193
B	1.2319201228	-0.5576583722	-1.5468045906
B	-2.7523733342	-0.8996001713	0.9697194981
B	3.016658117	-0.1027811088	-1.1461605807
B	-1.2400141513	1.7445917321	1.4436704744
B	3.049047906	1.5659105663	-0.5531729496
B	0.3225069435	1.3617489814	0.549004189
B	0.1707780772	-0.3826495272	0.1458020052
B	-1.4849788222	-1.3274288163	-0.3696920842
B	3.1822110825	0.3423860921	0.7162282723
B	-2.8477407697	1.0325912079	0.7743895487
B	2.1276445639	1.8428387082	0.9476993491
H	2.0321787978	1.3647384407	-2.9746467429
H	-2.3108608433	0.1285687728	3.2013560545
H	1.1391743536	3.3410629713	-0.819290293
H	0.4688079377	0.2875641617	2.7933114297
H	-0.6695298417	1.116593612	-1.8526283723
H	-0.6607664312	-2.1473252962	1.8451880017
H	0.9572851713	-1.3862347855	-2.3379189613
H	-3.618864607	-1.6019512449	1.3555595312
H	3.9033633807	-0.6628310184	-1.6861434946
H	-1.333046475	2.7092434044	2.1250614944
H	3.9800774516	2.2335340892	-0.8537064393
H	-1.3639018561	-2.3313450799	-0.9819032863
H	4.1751557588	0.0868255159	1.3023934593
H	-3.8424407656	1.6344448943	0.9868590669
H	2.4197501243	2.704222282	1.7061985271
H	-1.8308085053	1.9214848301	0.3181458719
H	2.2593603853	0.747556068	1.6228718235

Table S23. DFT/B3LYP calculated Cartesian coordinates for $[SeB_{18}H_{19}]^{-}$ anion **F**

B	-2.1740056003	-0.8203735011	-1.339578814
H	-2.624334398	-1.403585784	-2.2733628456
B	-1.1627949326	-1.6429249255	-0.1251202256
B	-0.44604036	-0.4816232005	-1.2829066329
B	-1.5711019608	0.8507867028	-1.5338755279
B	-3.1656527603	0.4055506164	-0.5888352268
B	-2.815759465	-1.0853309321	0.3100010625
H	-0.9474547379	-2.811231632	-0.1822779368
H	0.1766793871	-0.8285558356	-2.2371847136
H	-1.6654889614	1.4585799633	-2.548105881
H	-4.2451567108	0.6902712411	-0.9865036844
H	-3.7091996524	-1.8532074983	0.4800365513
B	0.134750677	-0.5706620209	0.4427384528
B	0.0562347321	0.9620539634	-0.5155967431
B	-2.6891359655	0.383477696	1.27422747
B	-1.4569287117	-0.9611971819	1.4564289042
H	-3.4664859131	0.6836462283	2.1189508825
H	-1.4681627057	0.253152851	1.8568230099
H	-1.428980798	-1.6341377914	2.4366504805
B	1.6674491753	-0.494057192	1.3962001919
B	0.7434886459	1.0023478915	1.1218233624
B	1.4549025691	1.8403453512	-0.3003806393
B	1.9255287033	-1.6635764661	0.1531719118
H	1.6456470575	-0.9435789434	2.5011101494
H	0.2036682182	1.5265269328	2.0474303674
B	2.4499163166	1.0718017863	0.9240727389
B	3.2039033818	-0.4897754129	0.4505908042
H	1.5251910578	3.0339922446	-0.3840861931
B	2.9108753684	0.822779734	-0.7957649713
H	1.9292987228	-2.8322546885	0.3726881324
H	3.1329600421	1.7272012632	1.653599201
H	4.2437147735	-0.7646294174	0.9659212056
H	3.8250145368	1.3875027743	-1.316601831
Se	-2.099269482	2.061642097	0.1277632084
H	3.6043627994	-1.5320589463	-1.9090063594
H	2.338353787	0.0945789314	-1.751077266
H	1.7256741627	-1.567535758	-1.1584586995
B	2.8820122611	-0.9539001606	-1.1630795555

Table S24. DFT/B3LYP calculated Cartesian coordinates for $SeB_{18}H_{20}$ **G**

B	-1.5726475139	1.6260318201	1.2841117093
H	-1.9109491577	2.326820542	2.1789874073
B	-0.4059562056	2.1653497019	0.0499452628
B	0.0563015844	0.9350634151	1.2686295782
B	-1.3369331332	-0.0908802254	1.6053356758
B	-2.7513824444	0.5557380146	0.5627915618
B	-2.1306306639	1.913417326	-0.3938350748
H	0.052171011	3.2588815981	0.0620929838
H	0.7463301069	1.1549752496	2.2065340284
H	-1.5168058608	-0.6504585973	2.6308691751
H	-3.8722116638	0.4308603929	0.9156537662
H	-2.8700709406	2.8157635907	-0.611909585
B	0.6753167296	0.8519029689	-0.4424149133
B	0.1299398502	-0.6321795704	0.4907885939
B	-2.2611627423	0.4065745611	-1.2921644236
B	-0.8069898261	1.4985172463	-1.5128764363
H	-3.0769044876	0.1758314281	-2.1175617394
H	-1.0480648529	0.2905376208	-1.8798274506
H	-0.6343207658	2.1077998005	-2.5152813113
B	2.1405503396	0.427103083	-1.3729138058
B	0.9388980832	-0.8510404497	-1.0559996173
B	1.4989365854	-1.8238116718	0.337150053
B	2.6105597517	1.5280127559	-0.1161270087
H	2.1893528993	0.8437287665	-2.4838554607
H	0.3440948383	-1.3148120104	-1.971368028
B	2.6333384916	-1.2131506971	-0.8681332372
B	3.679077912	0.1539612417	-0.4667635436
H	1.2901758971	-2.9882857468	0.4245656518
B	3.2515449332	-1.1037356143	0.7734085056
H	2.8083055203	2.6785976243	-0.3159629197
H	3.070846272	-2.0726331957	-1.5614936778
H	4.7241857178	0.2348535566	-1.0231981577
H	4.0351137762	-1.8622251198	1.2391229622
Se	-1.9450267456	-1.3143070646	-0.0506282786
H	4.2845340475	1.0876587905	1.9184628594
H	2.8356296933	-0.3424528448	1.7706394865
H	2.4426810973	1.4385250803	1.1936700117
B	3.4845426619	0.6495316869	1.1634808256
H	0.9634312044	-1.2797020541	1.3774075713

Table S25. DFT/B3LYP calculated Cartesian coordinates for $SeB_{17}H_{19}$ **H**

B	0.9221920818	0.7300697786	1.7299807368
B	-0.240314658	1.5969818879	0.650749919
B	1.4841391892	1.9752050188	0.5092097734
B	2.5565555431	0.6672310492	1.0231060033
B	-0.3498508593	-0.1741151176	0.8977494682
B	0.5051558815	1.7807940499	-0.9563626592
B	2.3141914998	1.1664758884	-0.7645210398
B	1.447179285	-0.818351145	1.204167525
H	0.7856639608	0.9105073157	2.8960037161
H	-0.9418388085	2.4026698487	1.1631960295
H	1.7878483349	3.0948322302	0.7644842322
H	-1.3079748375	-0.3148371844	1.8285429084
H	0.2944766264	2.697390236	-1.6773189616
H	3.1380625575	1.6479252396	-1.4657880483
H	1.5749803314	-1.7647066163	1.9027101438
B	-2.0468039023	-0.3574197588	-1.4782860403
B	-0.8912768547	-1.216601769	-0.4114369697
B	-2.6129353336	-1.5663938109	-0.2787721009
B	-3.699325337	-0.2416059038	-0.7752458031
B	-0.8005187134	0.5689452286	-0.6659500566
B	-1.6772987568	-1.3936242938	1.1935893206
B	-3.5178945747	-0.7135376186	0.9523488929
B	-3.7414402103	0.9899403869	0.4279295929
B	-2.6754563966	1.1944501077	-0.9804002472
H	-1.9308386061	-0.5117944594	-2.6499862329
H	-0.1844856893	-2.0293780343	-0.8980006034
H	-2.9569430832	-2.6813449327	-0.4996705264
H	0.2255700296	0.6854382798	-1.5732055393
H	-1.4938613823	-2.3020527617	1.9307698114
H	-4.3639728877	-1.2120418151	1.6156027006
H	-3.2277670725	0.4315551048	1.5298597162
H	-4.6157399841	1.713304658	0.7637661619
H	-2.6672067745	1.73209238	0.2200873569
H	-2.8244119023	2.0968332771	-1.7328875314
H	3.5515192615	0.8373709095	1.6388224935
H	-4.6434674619	-0.4991821382	-1.4451158161
Se	2.5563015039	-0.8238525155	-0.4685413259

Table S26. DFT/B3LYP calculated Cartesian coordinates for $[Se_3B_{18}H_{21}]^-$ anion **I**

H	-5.5616551844	0.7682039558	1.4516262504
H	-2.822953034	-0.696574134	2.5080576522
H	-3.0695554292	2.2680878652	2.324088
H	-5.3614070251	0.4460282095	-1.6950971295
H	-4.6225563042	2.9957358458	-0.3253789523
H	4.168589787	-2.8481163089	1.3608745867
H	-0.5977105512	1.3821885537	1.6722757624
H	1.602329618	-2.6704079339	-0.1865785384
H	2.8487804249	0.2384973687	1.7269291706
H	-1.8111273664	3.4123912872	-0.0427875457
H	-2.2341296648	0.516980664	-1.7605856324
H	4.2508145233	-2.6175171835	-1.5971092121
H	-2.9949876749	2.1971285947	-2.5706111391
H	6.1991670096	-1.1894004823	0.0586170873
H	-0.3439961889	1.5873338556	-1.6349539243
H	2.0171551891	-0.962457527	-2.570393255
H	2.942577203	0.4950194083	-1.5554774739
H	4.8149367055	0.2449129043	-2.4155177784
B	-4.5407548337	0.7327265965	0.8502098481
B	-2.9721825993	-0.0917295541	1.4995311848
B	-3.0577343835	1.6039544652	1.3359972267
B	-4.4032636394	0.5773887067	-1.005037302
B	-3.9458207788	2.0257179017	-0.1833571851
B	-1.4282190086	1.0126692907	0.9086135432
B	2.4052813765	-0.9345139688	1.4703135281
B	3.8202673359	-1.8303119791	0.8493523012
B	-2.1990478483	2.2886720285	-0.1031496127
B	2.3082796454	-1.7123450498	-0.0919264527
B	4.1166706274	-0.2597878754	1.598204099
B	-2.9383541067	1.5713581547	-1.5592680421
B	1.4371713951	-0.2351675209	-0.0022843556
B	-1.2374561492	1.128812809	-1.0053122247
B	3.8708697614	-1.6895004945	-0.9542831883
B	5.0543468478	-0.8762899186	0.0511206583
B	2.4907281985	-0.7015769805	-1.5084922009
B	4.2037919049	-0.0231671143	-1.4328575273
Se	-3.9544680334	-1.1247273289	0.0538771725
Se	-0.595052362	-0.6568192412	-0.1012267021
Se	4.7629186659	1.2052386451	0.2044982318
H	1.8729543814	-1.353442529	2.4505013219
H	4.6696421635	-0.1490565581	2.6435229086
H	1.612582812	0.9369952022	0.0947962606

Table S27. DFT/B3LYP calculated Cartesian coordinates for $[SeB_{10}H_{11}]^-$ anion **J**

B	-0.1773471998	0.8284426807	1.3026779437
B	-0.1881845163	1.6097664844	-0.4452643928
B	-0.151647671	-1.0770375326	1.1358324603
B	1.280013103	-0.0922417235	1.4588753028
B	1.2777109215	1.4245629659	0.5045944582
H	-0.7698434058	1.3733900338	2.1754048332
B	1.3541129381	0.9952802423	-1.2412250409
B	1.3586462672	-0.8364747547	-1.3454665268
B	-0.0987695498	-1.4917291442	-0.7169894851
B	1.342519745	-1.4776315232	0.3248380842
B	2.225939531	-0.0147822379	-0.0670291185
H	-0.7428178231	-1.7104177433	1.9498517315
H	1.7918349058	2.4083498772	0.9452034652
H	1.7766173126	-0.1216543322	2.5438246411
H	0.1364025604	1.0296989286	-1.6328524494
H	-0.7271999747	2.6562259471	-0.6123937749
H	3.4175305372	0.0906354328	-0.0447285073
H	1.8609499833	-2.5093091987	0.6400092159
H	-0.6897927955	-2.4825618506	-1.0309603301
H	2.0040205965	-1.3879901945	-2.1964466585
H	1.9575311478	1.728137454	-1.9694322331
Se	-1.5311896135	-0.0128078113	-0.1607346188

Table S28. DFT/B3LYP calculated Cartesian coordinates for $SeB_{10}H_{12}$ **K**

B	-1.0579497914	0.6743753475	0.9365809817
B	0.5783279907	-0.0994148138	1.5730546949
B	-1.0579497914	0.6743753475	-0.9365809817
B	-1.8660139783	-0.5566394291	0.
B	-0.9284721697	-1.0042819644	1.4677041333
H	-1.5461269637	1.5558165038	1.5548314806
B	0.5748154381	-1.8357333209	0.9553435561
B	0.5748154381	-1.8357333209	-0.9553435561
B	0.5783279907	-0.0994148138	-1.5730546949
B	-0.9284721697	-1.0042819644	-1.4677041333
B	-0.8791220162	-2.0252483852	0.
H	-1.5461269637	1.5558165038	-1.5548314806
H	-1.4960454843	-1.3191111088	2.4622048642
H	-3.052652241	-0.576761809	0.
H	1.4786986582	-0.9132166487	0.9792598524
H	1.0717946831	0.2894307972	2.5757155894
H	-1.3446261482	-3.1178178659	0.
H	-1.4960454843	-1.3191111088	-2.4622048642
H	1.0717946831	0.2894307972	-2.5757155894
H	1.0253067604	-2.7217089103	-1.6036540992
H	1.0253067604	-2.7217089103	1.6036540992
Se	0.7569411359	1.3595457192	0.
H	1.4786986582	-0.9132166487	-0.9792598524

Table S29. DFT/B3LYP calculated Cartesian coordinates for $SeB_{11}H_{11}$ **L**

B	-0.1773471998	0.8284426807	1.3026779437
B	-0.1881845163	1.6097664844	-0.4452643928
B	-0.151647671	-1.0770375326	1.1358324603
B	1.280013103	-0.0922417235	1.4588753028
B	1.2777109215	1.4245629659	0.5045944582
H	-0.7698434058	1.3733900338	2.1754048332
B	1.3541129381	0.9952802423	-1.2412250409
B	1.3586462672	-0.8364747547	-1.3454665268
B	-0.0987695498	-1.4917291442	-0.7169894851
B	1.342519745	-1.4776315232	0.3248380842
B	2.225939531	-0.0147822379	-0.0670291185
H	-0.7428178231	-1.7104177433	1.9498517315
H	1.7918349058	2.4083498772	0.9452034652
H	1.7766173126	-0.1216543322	2.5438246411
H	0.1364025604	1.0296989286	-1.6328524494
H	-0.7271999747	2.6562259471	-0.6123937749
H	3.4175305372	0.0906354328	-0.0447285073
H	1.8609499833	-2.5093091987	0.6400092159
H	-0.6897927955	-2.4825618506	-1.0309603301
H	2.0040205965	-1.3879901945	-2.1964466585
H	1.9575311478	1.728137454	-1.9694322331
Se	-1.5311896135	-0.0128078113	-0.1607346188

Table S30. DFT/B3LYP calculated Cartesian coordinates for $SeB_{11}Cl_{11}$ **M**

Se	-0.0008782942	0.0001266686	-2.1451784374
B	-0.9773693629	1.3450482054	-0.7119394161
B	0.9776663657	1.3452666209	-0.711798724
B	1.581596095	-0.5135534728	-0.711608553
B	0.0000229151	1.5474504557	0.7622674139
B	-1.4719713572	0.4782801401	0.7624236241
B	-0.9096727197	-1.2522534446	0.762216898
B	1.4716626272	0.4782216895	0.7625211461
B	0.9094539371	-1.2519764436	0.7623763265
B	-0.0002660637	-0.0000885947	1.6914418474
B	0.0001834049	-1.6623266155	-0.7122717946
B	-1.5813044879	-0.5138663333	-0.7116285665
Cl	-2.980331296	0.968617473	1.5804069763
Cl	0.0001662214	3.1334908711	1.5805710913
Cl	-1.9058290348	2.6225658473	-1.4940819232
Cl	1.9058702167	2.6226684649	-1.4939933351
Cl	3.0840043708	-1.0021292264	-1.4924139915
Cl	0.0013289126	-3.2412770041	-1.4950812324
Cl	1.8415859811	-2.5350853689	1.580686605
Cl	-1.8416102285	-2.5357692184	1.579953498
Cl	-0.0005201213	-0.0000424343	3.4703083193
Cl	-3.0836224546	-1.0016491151	-1.4923587074
Cl	2.9798343731	0.9682808352	1.5811536352

Table S31. DFT/B3LYP calculated Cartesian coordinates for SeB_5Cl_5 **N**

B	1.080330297	-0.6980166874	-1.475596533
B	-0.2558226517	-0.4057374288	-0.3102240712
B	0.434500505	-1.9476626857	-0.5174843759
B	0.7042919476	-1.1940633567	0.9859470041
B	2.038360821	-1.4849948845	-0.1773696553
Cl	-0.2722616931	-3.5046326537	-0.9382372615
Cl	3.6264310208	-2.2332708635	-0.0865226991
Cl	-1.8463724185	0.3374364639	-0.4049272385
Cl	1.3362904361	-0.3588288863	-3.1814457965
Cl	0.4417134536	-1.5393234139	2.6893903388
Se	1.5743529722	0.5640788765	0.1613425981

Table S32. DFT/ mPW1PW91 calculated Cartesian coordinates for $Se_2B_{18}H_{20}$ Compound **A**

B	-2.8145775034	0.9106048055	-1.0817848048
B	-0.5628279303	0.1546005166	1.4136398243
B	1.842447178	1.7866260683	0.9391614906
B	-1.0018643686	1.8601952637	0.9083766694
B	3.6323874282	-0.0686999304	-0.5147382838
B	-2.1942336332	-0.7882175094	1.2695144098
B	2.2143479056	-0.7567816642	-1.477080809
B	0.6493747805	-0.7107976465	-0.4126668238
B	3.2677618029	1.5988768028	-0.1011738007
B	-2.2008428525	0.9255697343	1.7487648776
B	-1.3088207877	1.9177189028	-0.7953497338
B	2.7389806557	0.9113486404	-1.648606434
B	0.988456796	0.5126412881	-1.5988859864
H	-3.5184440589	1.2079794156	-1.9898431728
H	-1.1094345139	2.8480435435	-1.5020031179
H	4.155666716	2.3870530919	-0.0571290352
H	-2.4749128499	1.2139016109	2.8682175646
H	-2.4609489731	-1.6327380771	2.0597658732
H	2.3681702096	-1.6395932187	-2.250828718
H	4.7194943502	-0.5270771788	-0.6142327356
H	0.0665587642	-0.0778930282	2.3957516389
H	3.2420678169	1.2217859573	-2.6784684592
H	0.3732935768	0.565390977	-2.6134107654
H	1.3898537287	3.0704638242	-1.1533961862
B	-3.4852698581	0.425877334	0.6535260174
Se	-2.9781961164	-1.112644482	-0.622308586
H	-0.5368152542	2.7924812401	1.4710911027
H	-0.0976323452	-1.6001009217	-0.6506838909
H	-4.6142233787	0.4084414507	1.0160604745
H	-0.9289380319	-1.0093865719	0.9473444107
H	-1.5455226486	0.8944107915	-1.5497428688
B	-2.6627211767	1.9436590493	0.3624005688
H	-3.287028147	2.9420642966	0.5198055809
Se	2.3504229645	-1.4305128358	0.5301053091
B	3.0385988431	0.4574408054	1.2197095113
B	0.2917851015	1.057553379	-0.0271028522
B	1.647535872	1.9779954974	-0.7686304786
H	1.7444254608	2.7006165573	1.6871139755
H	3.7591921001	0.3456761882	2.1539220575
H	1.8283883773	0.7444710321	1.711052187

Table S33. DFT/ mPW1PW91 calculated Cartesian coordinates for $[Se_2B_{18}H_{19}]^-$ anion **B**.

B	-2.7766398639	1.0872038781	1.003953117
B	-0.6278896105	-1.6782347214	-0.2095757955
B	1.7620692344	-0.0748852068	-1.6531573512
B	0.6142839393	-0.4808201124	-0.2097749073
B	-0.3950995689	-0.5820546452	1.3708396656
B	3.423165875	0.4144524805	-0.9076509775
B	-2.397810058	-1.4873797918	-0.6081763506
B	2.2397334701	1.5975527877	-1.4023677428
B	0.5883796836	1.0758569142	-1.0436202549
B	3.3453505663	0.2957685787	0.9852410182
B	-1.8650380768	-1.6775086253	1.0600258678
B	0.4398852712	0.9415164866	0.7236571373
B	-1.0554449198	1.1743619949	1.5926542799
B	3.1441793584	1.813671253	0.1331702804
B	1.3855170199	2.2282286622	0.0357808554
B	2.0479418211	1.4658595462	1.4790045519
H	-3.5765386037	1.8633041069	1.4234361663
H	-0.9340207752	1.9032428274	2.533076204
H	4.3188119614	0.0053708485	1.6018263581
H	-2.004764741	-2.7098372748	1.6431889645
H	-2.9569067872	-2.3585629626	-1.1973724567
H	2.4967383256	2.2655295268	-2.3548366535
H	4.4419546105	0.1584961509	-1.461074364
H	-0.2436807043	-2.7984676785	-0.39317051
H	4.0038183264	2.6364598312	0.206140112
H	1.0664007295	3.373715067	0.1356876102
H	2.1381721148	2.0803040398	2.4974933653
H	2.225186525	0.2372328212	1.7842075767
B	-3.303758467	-0.7163276589	0.8557538859
Se	-3.2994928293	0.3865242374	-0.9143876587
H	1.7360577735	-0.6567692463	-2.6881908715
H	0.280883549	-1.0528145567	2.243632964
H	-0.3107893617	1.3655424809	-1.772434217
H	-4.3739312361	-1.1018798468	1.2037563767
H	-1.2301925009	-1.2436334836	-1.2697528681
H	-1.6650250072	1.7883485377	0.6328969324
Se	2.5358302698	-1.330904604	-0.0879240442
B	-2.0972822011	-0.1978157153	1.9943574234
H	-2.383491112	-0.3278739275	3.1460693108

Table S34. DFT// mPW1PW calculated Cartesian coordinates for $[Se_2B_{17}H_{18}]^-$ anion **C**

B	-0.0112492621	2.6104067559	-0.0932764637
B	0.0112492621	-2.6104067559	-0.0932764637
B	0.362724094	1.6579352838	1.3479306934
B	-0.362724094	-1.6579352838	1.3479306934
B	-1.0036581842	2.7861073186	1.4105909421
B	1.0036581842	-2.7861073186	1.4105909421
B	-1.6961296005	3.0712961322	-0.1892102566
B	1.6961296005	-3.0712961322	-0.1892102566
B	0.4238457169	0.8507855765	-0.3025913465
B	-0.4238457169	-0.8507855765	-0.3025913465
B	0.	0.	1.1915997665
B	-1.1471006052	1.1776928748	2.1001099078
B	1.1471006052	-1.1776928748	2.1001099078
B	-2.510593825	1.9081560779	1.1185023865
B	2.510593825	-1.9081560779	1.1185023865
B	-0.8909399323	1.7851103689	-1.309358126
B	0.8909399323	-1.7851103689	-1.309358126
H	0.8311353273	3.3823396133	-0.4386374465
H	-0.8311353273	-3.3823396133	-0.4386374465
H	1.2988954852	1.9205849361	2.0396348262
H	-1.2988954852	-1.9205849361	2.0396348262
H	-0.9247255943	3.7444939038	2.1148459352
H	0.9247255943	-3.7444939038	2.1148459352
H	-2.0838274368	4.1547897615	-0.4882897118
H	2.0838274368	-4.1547897615	-0.4882897118
H	1.5081699132	0.8564248105	-0.7905568786
H	-1.5081699132	-0.8564248105	-0.7905568786
H	-1.3288321311	1.0375943298	3.269075837
H	1.3288321311	-1.0375943298	3.269075837
H	-3.5240616129	2.1624607423	1.6869621936
H	3.5240616129	-2.1624607423	1.6869621936
H	-0.6968764717	1.9412824142	-2.474231585
H	0.6968764717	-1.9412824142	-2.474231585
H	-2.0576025096	0.5907156443	1.4298375979
H	2.0576025096	-0.5907156443	1.4298375979
Se	-2.8375843408	1.4800078211	-0.8723440739
Se	2.8375843408	-1.4800078211	-0.8723440739

Table S35. DFT/ mPW1PW91 calculated Cartesian coordinates for $Se_2B_{17}H_{17}$ **D**

Se	2.0231876271	-1.4090736801	0.2655782363
Se	-2.9342596814	-0.0953179705	-0.957829729
B	1.8690063005	1.0372649985	-1.8300884908
B	-1.9118835261	0.2056371494	2.0725864254
B	1.3357096026	2.1944248524	-0.5847469192
B	-0.1920241157	0.3429934181	1.8166812081
B	0.2436175346	0.9017604473	-1.138670466
B	-1.0140447174	-1.1619931977	1.2728221357
B	1.2083093079	-0.5523384838	-1.5084566294
B	-2.720524935	-0.8818983631	0.9444814709
B	2.9790681918	-0.1045727407	-1.1182750834
B	-1.2270691008	1.7268119062	1.4508161463
B	3.0187626455	1.5629161396	-0.5552892813
B	0.3225224514	1.3674682702	0.546976872
B	0.1697878495	-0.3644827872	0.1482628181
B	-1.453969226	-1.2993176937	-0.3697098708
B	3.1373197031	0.3503260066	0.712130453
B	-2.7991982739	1.0255678403	0.7731879251
B	2.1088001441	1.8369831831	0.9369482825
H	2.0129931141	1.3384803479	-2.9699151788
H	-2.2902552743	0.1117766179	3.1948179011
H	1.1273174667	3.3409614508	-0.8192824911
H	0.4950273633	0.2860867294	2.7857459064
H	-0.6764865041	1.1238747474	-1.8512659194
H	-0.6325891467	-2.1437443329	1.8268114315
H	0.9315274479	-1.3856960216	-2.3004625081
H	-3.5913448948	-1.5849167906	1.3336628582
H	3.8659443134	-0.6712324637	-1.6606167122
H	-1.3342956018	2.7059590217	2.1150344095
H	3.9627308556	2.2193788538	-0.8547049699
H	-1.3315635439	-2.303638712	-0.9880823537
H	4.1227351329	0.0851463818	1.3147187207
H	-3.7959814834	1.6356698064	0.9754687172
H	2.4041945272	2.6874134217	1.7117228707
H	-1.7697619423	1.8701986622	0.2974868498
H	2.1849003886	0.7312189855	1.5930029645

Table S36. DFT/ mPW1PW91 calculated Cartesian coordinates for $[SeB_{18}H_{21}]^{-}$ anion **E**

B	-2.3909551225	-1.440815476	-0.7083137532
B	1.5526801811	1.1065703685	1.6299530814
B	-1.5058269714	-0.1232245546	-1.4836654859
B	0.3747564524	1.9178998884	0.6178471806
B	-3.2588223801	-0.028480912	-1.3283380926
B	2.0802439792	1.9873507184	0.1478087374
B	-3.9148147328	-0.9254962227	0.0607854128
B	2.9490092499	0.5596826181	0.7439630206
B	-0.8485931947	-0.9130241307	0.0352479843
B	-0.0323894813	0.2758131166	1.295320408
B	-0.671824811	0.8675060715	-0.2951932389
B	-2.3067216105	1.3811799442	-0.9726755709
B	0.8297897152	1.7475591114	-1.0639169842
B	-3.8595313256	0.8720047895	0.0519048064
B	2.3781135709	0.7748230023	-1.1023466446
B	-2.4808127858	-1.3985560024	1.0225027043
B	1.6116957233	-0.5933226888	1.3850334235
H	-2.355188683	-2.5198651488	-1.2145193411
H	1.6831801148	1.5337127821	2.7364656991
H	-1.0356348935	-0.2803146869	-2.5681394278
H	-0.1487502214	2.9344615895	0.9604475216
H	-3.9072004313	0.0339241242	-2.3272719629
H	2.6587233177	3.0279865407	0.2044139731
H	-4.9095715197	-1.5702497233	-0.0606580284
H	4.0705959133	0.6663180902	1.1247715621
H	-0.1037217941	-1.7977013884	-0.2233190922
H	-0.6501223681	0.3442726958	2.3158978404
H	-2.3482260844	2.3675876659	-1.6383663131
H	0.6474649409	2.5664466383	-1.9089234364
H	-4.8179033089	1.5772792294	0.0089974099
H	3.132079125	0.9592201919	-2.0040996757
H	-2.4036173932	-2.3565911112	1.7257129572
H	1.8146228071	-1.37348715	2.2633048786
H	-1.7474990153	1.6624012522	0.129445999
H	1.0747892703	0.6224032792	-1.6145746488
B	-3.5873058407	-0.1083414267	1.5307700441
Se	2.5185710922	-1.1434439338	-0.3536422055
H	-2.3345530848	-0.3553761844	1.8289188021
H	-4.2329185631	-0.083222502	2.5275160508
H	-3.2904098362	1.1449095343	1.2214344048

Table S37. DFT/ mPW1PW91 calculated Cartesian coordinates for $[SeB_{18}H_{19}]^{-}$ anion **F**

B	-2.1576978688	-0.8426775975	-1.3419705528
H	-2.6190079231	-1.4262792581	-2.2713963229
B	-1.1478989996	-1.6591909821	-0.1280805164
B	-0.4302810709	-0.5026917768	-1.2776942452
B	-1.5523135776	0.8069706446	-1.5683817636
B	-3.1140646555	0.4096769711	-0.5933454338
B	-2.7944034918	-1.0827412619	0.3064814605
H	-0.9374650982	-2.8303725146	-0.1773368221
H	0.2086684069	-0.8519504116	-2.2217231125
H	-1.6414794695	1.4224668156	-2.5799341952
H	-4.1892322371	0.7438733219	-0.9685544382
H	-3.7053584184	-1.8288045418	0.4869705701
B	0.1353910053	-0.5804925405	0.4466684217
B	-0.0000546471	0.9752868637	-0.4825830665
B	-2.6380381082	0.3871159983	1.2538348882
B	-1.4393782396	-0.969007775	1.4486416061
H	-3.4169388295	0.7246509721	2.0854439579
H	-1.421320623	0.2510008755	1.8501788784
H	-1.4252930223	-1.6363784564	2.4346959689
B	1.6606254606	-0.4856722777	1.4054590203
B	0.7345869825	0.9976144099	1.1374101832
B	1.4276789754	1.8381806161	-0.2788130439
B	1.8926292816	-1.6452716988	0.1474286493
H	1.6371197551	-0.9493734363	2.5057334522
H	0.1883340239	1.5153217585	2.0675826688
B	2.4351033016	1.0714374368	0.9297838602
B	3.1798679709	-0.4832256005	0.4470352659
H	1.4925860447	3.0354219728	-0.343284191
B	2.8714819112	0.8308340787	-0.7899736459
H	1.8938135107	-2.8167552616	0.36249923
H	3.1197879286	1.7280906975	1.658288569
H	4.2268657777	-0.7650811308	0.9462240201
H	3.7855628155	1.3916625993	-1.3192283867
Se	-1.9717653497	2.0163654639	0.1049868341
H	3.5573140169	-1.503746026	-1.9226403424
H	2.2683095086	0.117362355	-1.7346675079
H	1.6879753495	-1.5481041094	-1.1651827777
B	2.8419628629	-0.9334762138	-1.1621327999

Table S38. DFT/ mPW1PW91 calculated Cartesian coordinates for $SeB_{18}H_{20}$ **G**

B	-1.5545704369	1.6254782515	1.2875375414
H	-1.8977983373	2.321196715	2.1859375405
B	-0.3894601294	2.1687839384	0.0614731881
B	0.07114592	0.9359247639	1.2699613648
B	-1.3189710828	-0.0893599125	1.6029239953
B	-2.7235653182	0.5497748029	0.5599536531
B	-2.1054404743	1.9116037996	-0.3862166283
H	0.0718789831	3.2626168526	0.0717249774
H	0.7634389167	1.1464440751	2.2114256673
H	-1.4996603336	-0.6557180389	2.6270316111
H	-3.8467225677	0.4156826855	0.9088354332
H	-2.8513986055	2.8077665627	-0.6127781245
B	0.681392176	0.8518485378	-0.4427417075
B	0.1267709178	-0.6282103483	0.4831554229
B	-2.22231194	0.4020000208	-1.2775484575
B	-0.782161419	1.4943461124	-1.4983560637
H	-3.0381910678	0.1679321418	-2.10498656
H	-1.005451754	0.2790566337	-1.8653250769
H	-0.6138925314	2.1028189557	-2.5037321258
B	2.1321286012	0.4163755015	-1.3875675566
B	0.9324607837	-0.8506301157	-1.0644996697
B	1.488550182	-1.8146034749	0.3289387749
B	2.5719285338	1.5197325432	-0.1231175067
H	2.1745800468	0.841142646	-2.4969621072
H	0.3241938303	-1.3164738779	-1.9739915509
B	2.6221615941	-1.2128865724	-0.8757622637
B	3.6554572819	0.15575521	-0.4711207329
H	1.2790063115	-2.9804250449	0.419268305
B	3.2190999917	-1.0917415552	0.7707082146
H	2.7681277118	2.6721167448	-0.325710518
H	3.0626347144	-2.0781174753	-1.5619700365
H	4.7092852611	0.2427402676	-1.0122706504
H	4.001453875	-1.8461844522	1.2492115861
Se	-1.90284256	-1.2926307496	-0.0487146368
H	4.2431484563	1.09394549	1.9123887738
H	2.7867212209	-0.3299494415	1.7646199177
H	2.402297648	1.4380222042	1.1885475319
B	3.447500633	0.652328876	1.1529972624
H	0.9628769667	-1.2721402733	1.3780642122

Table S39. DFT/ mPW1PW91 calculated Cartesian coordinates for $SeB_{17}H_{19}$ **H**

B	0.9083352633	0.7372984554	1.7298574362
B	-0.2538647066	1.5984612454	0.6576312785
B	1.4658580626	1.9739412398	0.5160981509
B	2.5352110415	0.6585761748	1.0121327689
B	-0.3595376471	-0.1682677136	0.8976044522
B	0.4874067694	1.7806650726	-0.9458705883
B	2.2781126713	1.160685468	-0.7656822536
B	1.4153011518	-0.8141956404	1.1940882663
H	0.7736986715	0.9116179452	2.8983085
H	-0.9588244235	2.406309014	1.1674537483
H	1.7756084154	3.0936538736	0.7685378401
H	-1.3144386243	-0.3052650634	1.8325544649
H	0.276834993	2.694806666	-1.6722338243
H	3.1028828223	1.6377988674	-1.472239245
H	1.5436606904	-1.7639664316	1.8914176441
B	-2.0372217303	-0.3672202255	-1.4813932301
B	-0.8833957663	-1.2145053534	-0.4128195844
B	-2.6015997508	-1.5675280566	-0.2854419796
B	-3.6801944851	-0.243000304	-0.776741766
B	-0.805341571	0.5696964276	-0.6621433197
B	-1.6722750513	-1.3866579193	1.1870871901
B	-3.4942101663	-0.7084731397	0.946834396
B	-3.7096184038	0.9862875876	0.425564408
B	-2.6495592101	1.1866076868	-0.9769508591
H	-1.9157944045	-0.5151127408	-2.6546202608
H	-0.1700238364	-2.0295692064	-0.8908806416
H	-2.9470777641	-2.6848756274	-0.4977153651
H	0.2282161495	0.677304682	-1.5659208833
H	-1.4886903484	-2.2941601879	1.9280644801
H	-4.3402039459	-1.2013867074	1.6171745347
H	-3.1923996344	0.433317648	1.5294854175
H	-4.5763539536	1.7178321547	0.7676735363
H	-2.6259352734	1.7226160216	0.2256954732
H	-2.7950557129	2.0937269047	-1.7270217728
H	3.5347395179	0.8259480427	1.6248326084
H	-4.632455248	-0.4906468359	-1.4407144163
Se	2.5104174379	-0.8131470241	-0.4685196042

Table S40. DFT/ mPW1PW91 calculated Cartesian coordinates for $[Se_3B_{18}H_{21}]^-$ anion **I**

H	-5.6660934961	0.6374804244	1.4550947901
H	-2.8974009089	-0.8135609711	2.4395438632
H	-3.2029590182	2.1517805704	2.3809956413
H	-5.4665383854	0.4414682651	-1.6961742398
H	-4.7740881417	2.9503047784	-0.2383449398
H	4.0312268898	-2.8393015634	1.2180663306
H	-0.7254961535	1.3204701614	1.6912780191
H	1.4366092881	-2.6515392906	-0.3017735498
H	2.6574365965	0.2197666098	1.6789809745
H	-1.9589429668	3.396364883	0.0478326656
H	-2.372963117	0.5379189028	-1.7497451559
H	4.0820561468	-2.5190318394	-1.735289678
H	-3.1278131643	2.2498418735	-2.5101145237
H	6.0179269061	-1.103788022	-0.0525527066
H	-0.4802182997	1.6248027797	-1.5930330437
H	1.8126747795	-0.8716834341	-2.6302996497
H	2.7166562108	0.5739383485	-1.5788691314
H	4.5880912828	0.3739506701	-2.4594507895
B	-4.6443946095	0.6420274456	0.850140099
B	-3.0555674582	-0.1714056172	1.4530400792
B	-3.1852542689	1.5297069747	1.3648738598
B	-4.506080939	0.562697528	-1.0040892615
B	-4.0787343708	1.9879252428	-0.1302596505
B	-1.5571219459	0.9698175783	0.9158107239
B	2.2413335488	-0.9577158798	1.3914885209
B	3.6583745476	-1.8155541118	0.7344465562
B	-2.3403439813	2.2715546541	-0.0443790012
B	2.1427414753	-1.6940004034	-0.1854897928
B	3.930900839	-0.2610993515	1.5203981568
B	-3.0666283392	1.5951080641	-1.5163108302
B	1.2708696138	-0.2207648748	-0.0420288029
B	-1.3757629903	1.1427081214	-0.9796880193
B	3.6899898248	-1.6210227991	-1.0565978717
B	4.865772607	-0.8106060841	-0.0408081566
B	2.2955004269	-0.63622299	-1.5651418309
B	3.9859832668	0.0648963719	-1.4807640675
Se	-4.0293970194	-1.1505129875	-0.0148168569
Se	-0.7379206315	-0.6500969136	-0.1366720366
Se	4.5231073573	1.235006642	0.1743920975
H	1.7213144455	-1.4131148889	2.3635993437
H	4.4960641365	-0.1705572215	2.5634651326
H	1.4484780158	0.9530063538	0.0885067327

Table S41. DFT/ mPW1PW91 calculated Cartesian coordinates for $[SeB_{10}H_{11}]^-$ anion **J**

B	-0.1772312272	0.8240270602	1.2943758412
B	-0.1842820144	1.5960301845	-0.443856342
B	-0.1532357694	-1.0676526405	1.1302128785
B	1.2826313342	-0.0933959815	1.4545041851
B	1.2802806941	1.4196436641	0.5057374686
H	-0.7704436706	1.3690060506	2.1694034302
B	1.3533864487	0.9955484354	-1.2328628832
B	1.3570812573	-0.8266210651	-1.3422881151
B	-0.1027384198	-1.4778564919	-0.7080262677
B	1.3412655037	-1.4718196323	0.319739103
B	2.2270745576	-0.0116047017	-0.0652508088
H	-0.7453853335	-1.7008618008	1.9465567248
H	1.788334936	2.4081969854	0.9448915966
H	1.7731893254	-0.124598566	2.5430740204
H	0.138139487	0.995055925	-1.6334934227
H	-0.7266388966	2.6429611419	-0.610786991
H	3.4197068282	0.0928467076	-0.0437511491
H	1.8546296946	-2.5078556356	0.6319456115
H	-0.6931113792	-2.4710539308	-1.0233269866
H	1.9973491884	-1.3809778988	-2.1963194371
H	1.9510793945	1.7325719312	-1.9630888047
Se	-1.5040449391	-0.0117377407	-0.1598006519

Table S42. DFT/ mPW1PW91 calculated Cartesian coordinates for $SeB_{10}H_{12}$ **K**

B	-1.0513854527	0.6707443498	0.9315743129
B	0.5740655878	-0.1003525462	1.5555483824
B	-1.0513854527	0.6707443498	-0.9315743129
B	-1.8657675384	-0.5589938348	0.
B	-0.9297903907	-1.0064561382	1.4613567645
H	-1.5378297668	1.5549566344	1.551280119
B	0.5710759436	-1.8288858525	0.9473136354
B	0.5710759436	-1.8288858525	-0.9473136354
B	0.5740655878	-0.1003525462	-1.5555483824
B	-0.9297903907	-1.0064561382	-1.4613567645
B	-0.8838407658	-2.024727744	0.
H	-1.5378297668	1.5549566344	-1.551280119
H	-1.4912992566	-1.3185335612	2.4612966953
H	-3.053492923	-0.5737001354	0.
H	1.482945925	-0.9109818949	0.9621664219
H	1.0679627505	0.2901601905	2.5598475069
H	-1.3448893902	-3.1204350765	0.
H	-1.4912992566	-1.3185335612	-2.4612966953
H	1.0679627505	0.2901601905	-2.5598475069
H	1.0242044421	-2.7123480114	-1.5996893493
H	1.0242044421	-2.7123480114	1.5996893493
Se	0.7433160572	1.3366404432	0.
H	1.482945925	-0.9109818949	-0.9621664219

Table S43. DFT/ mPW1PW91 calculated Cartesian coordinates for $SeB_{11}H_{11}$ **L**

Se	0.0000001621	0.0000751231	-1.4894451266
B	0.0000000178	1.6200208925	-0.1125967395
B	1.5408440814	0.5007348239	-0.1126352449
B	0.9523958846	-1.3106865682	-0.112673082
B	0.8937160956	1.2301669645	1.3503469661
B	-0.8937163387	1.2301667737	1.3503470811
B	-1.4462496338	-0.4698378688	1.350297025
B	1.4462494904	-0.4698376673	1.3502969707
B	-0.0000001595	-1.5206848119	1.3502512226
B	-0.0000000284	-0.0000854866	2.273643743
H	0.0000001262	-0.0000124163	3.4621483877
H	-0.0000001352	-2.5962768145	1.8560561245
H	-2.4692060994	-0.8022381361	1.8560734131
H	2.4692059371	-0.8022382358	1.8560731976
H	1.525883375	2.1004430103	1.8560274053
H	1.5530660177	-2.137389364	-0.7113097703
H	2.5126208297	0.816340106	-0.7115619114
H	0.0000000129	2.6418981331	-0.7112359863
B	-0.9523956986	-1.3106870515	-0.1126731097
H	-1.5530659171	-2.1373898093	-0.7113098151
H	-1.525883388	2.1004429074	1.8560276446
B	-1.5408439151	0.5007350113	-0.112635325
H	-2.5126207207	0.816340496	-0.7115617107

Table S44. DFT/ mPW1PW91 calculated Cartesian coordinates for $SeB_{11}Cl_{11}$ **M**

Se	0.0000780297	0.0003846585	-2.1165693595
B	-0.9692574606	1.3341340816	-0.7102192095
B	0.9697273461	1.3347049013	-0.7091203427
B	1.5683077641	-0.5094422379	-0.7101332144
B	-0.0003036393	1.5398704452	0.7656400015
B	-1.4645986081	0.4758159803	0.7652712874
B	-0.9049752692	-1.2457214553	0.7657517932
B	1.4645270634	0.4756925043	0.7657608712
B	0.9052021459	-1.2457017425	0.7654962066
B	-0.0000921436	0.0000309229	1.6922344626
B	-0.0001784641	-1.6490400808	-0.709723519
B	-1.5682669364	-0.5098343415	-0.7098235003
Cl	-2.9653498569	0.9634477739	1.5696421909
Cl	-0.0009253715	3.1177351346	1.5703462063
Cl	-1.8906272527	2.60163043	-1.4938742617
Cl	1.8922255552	2.6037601906	-1.4886112369
Cl	3.0582726043	-0.9944670021	-1.4942125356
Cl	-0.0001181546	-3.2170332067	-1.4916633821
Cl	1.8329637849	-2.5220393438	1.5700854548
Cl	-1.8323024632	-2.5221882233	1.570520585
Cl	-0.0003695719	0.0000538333	3.4584599945
Cl	-3.0591465276	-0.9947968041	-1.4917251764
Cl	2.9652074257	0.9630035812	1.5704393838

Table S45. DFT/ mPW1PW91 calculated Cartesian coordinates for SeB_5Cl_5 **N**

B	1.0783319767	-0.6998976212	-1.4731061642
B	-0.2540964848	-0.4089369711	-0.3111990111
B	0.4323757974	-1.9534138088	-0.5184557489
B	0.7035494226	-1.1943426667	0.982269628
B	2.0346183144	-1.484932926	-0.1786522405
Cl	-0.2695268845	-3.4996890483	-0.9363424954
Cl	3.6169442867	-2.2204718673	-0.0856217004
Cl	-1.830943571	0.3385478189	-0.4023186031
Cl	1.3382233437	-0.3534301755	-3.1659801363
Cl	0.4472750474	-1.5286589532	2.6781311496
Se	1.5650634414	0.5402106992	0.1561476323
